# Phase Advancing Is a Common Property of Multiple Neuron Classes in the Mouse Retina

**DOI:** 10.1523/ENEURO.0270-22.2022

**Published:** 2022-09-01

**Authors:** Victor J. DePiero, Bart G. Borghuis

**Affiliations:** 1 Department of Anatomical Sciences and Neurobiology, University of Louisville School of Medicine, Louisville, KY 40202; 2 Department of Biology, University of Virginia, Charlottesville, VA 22904

**Keywords:** calcium imaging, electrophysiology, glutamate imaging, neural coding, object motion, Retina

## Abstract

Behavioral interactions with moving objects are challenged by response latencies within the sensory and motor nervous systems. In vision, the combined latency from phototransduction and synaptic transmission from the retina to central visual areas amounts to 50–100 ms, depending on stimulus conditions. Time required for generating appropriate motor output adds to this latency and further compounds the behavioral delay. Neuronal adaptations that help counter sensory latency within the retina have been demonstrated in some species, but how general these specializations are, and where in the circuitry they originate, remains unclear. To address this, we studied the timing of object motion-evoked responses at multiple signaling stages within the mouse retina using two-photon fluorescence calcium and glutamate imaging, targeted whole-cell electrophysiology, and computational modeling. We found that both ON-type and OFF-type ganglion cells, as well as the bipolar cells that innervate them, temporally advance the position encoding of a moving object and so help counter the inherent signaling delay in the retina. Model simulations show that this predictive capability is a direct consequence of the spatial extent of the cells’ linear visual receptive field, with no apparent specialized circuits that help predict beyond it.

## Significance Statement

Signal transduction and synaptic transmission within sensory signaling pathways costs time. Not a lot of time, just tens to a few hundred milliseconds depending on the sensory system, but enough to challenge fast behavioral interactions under dynamic stimulus conditions, like catching a moving fly. To counter neuronal delays, nervous systems of many species use anticipatory mechanisms. One such mechanism in the mammalian visual system helps predict the future position of a moving target through a process called phase advancing. Here, we ask for functionally diverse neuron populations in the mouse retina how common is phase advancing and demonstrate that it is common and generated at multiple signaling stages.

## Introduction

Vision is not real time. Phototransduction and the transmission of visual information across multiple synaptic stages to central visual areas cause a cumulative delay of tens to hundreds of milliseconds, depending on species and stimulus conditions (Baylor et al., 1984; Schnapf et al., 1987). As a consequence, visual perception and the visual information available for planning motor action lags the actual state of the external world. This lag challenges dynamic behavioral interactions with moving objects and with stationary objects during self-motion, where real-time knowledge of location is critical. Examples are myriad and range from prey capture (Borghuis and Leonardo, 2015; Mischiati et al., 2015; Wardill et al., 2017; Mearns et al., 2020; Johnson et al., 2021) to sports, including tennis and baseball, as well as object collision avoidance during self-motion, for example, during skiing, trail running, and mountain biking.

The biological relevance of accurate representation of visual object motion in vertebrate animals is evident from a range of known adaptations both within the retina (Zhang et al., 2012) and in downstream brain nuclei (Jancke et al., 2004; Mysore et al., 2010; Cafaro et al., 2020). Specific evidence that compensation for neuronal response latency occurs already at the level of the retina stems from electrophysiological recordings in goldfish, salamander, mouse, and rabbit (Berry et al., 1999; Leonardo and Meister, 2013; Trenholm et al., 2013a; Johnston and Lagnado, 2015). Multi-electrode array recordings showed that specific ganglion cell populations in salamander (ON-type and OFF-type ganglion cells) and rabbit retina (OFF-α-type ganglion cells) compensate for the phototransduction delay, either partially or completely, using a predictive mechanism called phase advancing (Berry et al., 1999; Leonardo and Meister, 2013). In these identified phase-advancing ganglion cells, a moving stimulus on a continuous trajectory evoked a population response that matched or preceded the leading edge of the moving spot or bar, thus compensating for the visual signaling delay. In the same cell populations, the action potential response to a stationary flashed spot lagged the stimulus by 50–100 ms, demonstrating that the delay compensation is motion-dependent. This predictive mechanism is evident during continuous object motion and additional mechanisms have been demonstrated that signal motion reversal (Schwartz et al., 2007).

Two different mechanisms reportedly contribute to the phase-advanced response in ganglion cells. The first, based on population ganglion cell recordings in salamander, is a gain-control mechanism that renders the ganglion cell response transient and shifts the population stimulus representation toward the leading edge of the stimulus (Berry et al., 1999; Leonardo and Meister, 2013). The second, based on electrophysiological whole-cell recordings in goldfish retinal ganglion cells (Johnston and Lagnado, 2015), is feed-forward inhibition from amacrine cells onto a subset of ganglion cell types (brisk transient, brisk sustained, and orientation-selective cells). Thus, studies in two different vertebrate model systems each demonstrated phase-advanced responses at the retinal ganglion cell level but, the mechanism claimed to generate it distinctly differed between them, i.e., cell-intrinsic gain control in salamander and amacrine cell inhibition in goldfish.

The goal of this study was to determine how general phase advancing is among identified ganglion cell types in the mouse retina, and to resolve its origin and the mechanisms that underlie it. We tested for phase advancing at three organizational levels in an *ex vivo* retinal whole-mount preparation: ganglion cell populations using two-photon imaging of visually evoked calcium responses; excitatory and inhibitory synaptic input of identified ganglion cell types using whole-cell voltage-clamp electrophysiology; and bipolar cell synaptic output using two-photon fluorescence glutamate imaging of the inner plexiform layer (IPL; Borghuis et al., 2013). To assess contributions of amacrine cell circuits we perturbed inhibitory signaling pharmacologically, and tested for nonlinear contributions to response timing at the ganglion cell level using model simulation to compare observed responses against responses predicted from the measured linear spatiotemporal receptive field.

Our data show that the majority of ganglion cell types in mouse temporally advance the position encoding of a moving object. The forward shift in response timing of these cell types is a direct consequence of the spatial extent of their visual receptive field and is apparent already at the level of the bipolar cell output. The observed advanced response onset time is the foundation for predictive position encoding at the ganglion cell population level and at subsequent signaling stages, potentially through cell-intrinsic mechanisms reported previously (Leonardo and Meister, 2013).

## Materials and Methods

### Animals and retinal preparation

All animal procedures were approved by the Institutional Animal Care and Use Committee at the University of Louisville School of Medicine and were in compliance with National Institutes of Health guidelines.

Experiments used mice of either sex, aged two to six months old and maintained on a C57BL6/J background. Two-photon fluorescence calcium imaging experiments used two strains of *Thy1*-GCaMP6f-WPRE transgenic mice, GP5.11 and GP5.17 (The Jackson Laboratory #025393 and #024339, respectively). Targeted whole-cell electrophysiology experiments used KCNG4-cre mice (Krieger et al., 2017) crossed with Cre-dependent fluorescence reporter line Ai3 (Jackson Laboratory #007903). Genotype positive offspring from this cross selectively expressed enhanced yellow fluorescent protein (EYFP) in four α-like ganglion cell types, ON and OFF sustained and transient (Pang et al., 2003; Van Wyk et al., 2009). Two-photon fluorescence glutamate imaging experiments used wild-type C57BL6/J mice and viral transduction through intraocular injection as described below.

Data were obtained from the ventral half of whole-mount mouse retinae recorded *in vitro*, as described previously (Borghuis et al., 2013; Fransen and Borghuis, 2017). Briefly, mice were dark adapted for ∼30 min, anesthetized with isoflurane and killed by cervical dislocation under dim red illumination. Eyes were enucleated and hemisected in oxygenated Ames medium (95%O_2_-5%CO_2_; Sigma-Aldrich) under infrared illumination using night-vision scopes (OWL Night Vision Scopes; B. E. Meyers) mounted on a dissecting microscope (Olympus SZ61). The retina was then radially incised for flattening, separated from the eyecup at the RPE layer, and mounted ganglion cell-side up on nitrocellulose filter paper discs (Millipore Sigma); 1.2 mm in diameter holes in the filter paper enabled visual stimulation of the photoreceptors through the condenser light path of the microscope. The paper disk with retina preparation was placed in a custom-designed, 3D-printed recording chamber and mounted onto the stage of a custom-built two-photon fluorescence microscope (Pologruto et al., 2003). The retina preparation was continuously perfused with nonrecycled oxygenated Ames medium at physiological temperature (∼6 ml/min; 33–35°C) for the duration of the experiment; approximately 3 h per retina.

### Viral transduction of ganglion cells

Intravitreal injections of 1.4–1.6 μl of AAV2/1-*hSynapsin*-iGluSnFR (Marvin et al., 2013) in suspension with a typical titer of ∼1 × 10^13^ IU/μl were performed in the left and right eyes of three- to four-week-old mice (C57BL6/J). For injections, isoflurane anesthesia was induced (2–3% in O_2_) in an induction chamber and maintained (1–1.2% in O_2_) using a nose cone on the stage of a dissecting microscope inside a fume hood. A topical anesthetic (Proparacaine hydrochloride ophthalmic solution, USP 5%; Henry Schein Medical) was applied to each eye. To inject, we used curved forceps to rotate the eye outward and stabilize it. We then used a 30-gauge hypodermic needle to puncture the corneal limbus at the cornea-scleral boundary and a modified Hamilton syringe (Borghuis Instruments) fitted with a 33-gauge, blunt-style tip curved to avoid damaging the lens, to inject the viral load over the ventral portion of the retina. Following 18–21 d of incubation, injected animals were killed and retinas of animals now considered young adult (six to eight weeks) harvested and prepared for recording as described above.

### Fluorescence imaging

Two-photon fluorescence imaging was performed with a modified Olympus microscope controlled with ScanImage 3.8 software and an Olympus 60×, 1.0 NA, LUMPlanFL/IR objective. The scan laser (Chameleon Ultra II; Coherent) was tuned to 910 nm for GCaMP6f, EYFP and iGluSnFR fluorescence excitation in retinal areas. The field of view during GCaMP6f imaging in the ganglion cell layer ranged from 35 × 35 to 42 × 42 μm, depending on optical zoom set to capture a cluster of 5–15 GCaMP6f-expressing ganglion cells. The field of view during iGluSnFR imaging of bipolar cell glutamate release in the IPL was 35 × 35 μm, approximating the axon terminal area of three to five bipolar cells (Tsukamoto and Omi, 2017).

Fluorescence responses were recorded with X-Y frame scans at 15 frames per second (fps). Stimulus-evoked bipolar cell glutamate release was recorded at two levels of the IPL, ∼16 and 31 μm distal to the ganglion cell layer, to record signaling onto the iGluSnFR-expressing dendrites of, respectively, ON-type and OFF-type ganglion cells, including α-type cells.

### Electrophysiology

Borosilicate glass microelectrodes were filled with intracellular solution containing (in mm): 120 Cs-methanesulfonate, 5 TEA-Cl, 10 HEPES, 10 BAPTA, 3 NaCl, 2 QX 314-Cl, 4 ATP-Mg, 0.4 GTP-Na2, and 10 phosphocreatine-Tris2 (pH 7.3, 280 mOsm), and a red fluorescent dye (Sulforhodamine 101; Sigma-Aldrich). Voltage-clamp recordings were performed at the reversal potential for chloride and cations, respectively, −67 and +15 mV. Membrane potential recordings were obtained in current clamp mode (I = 0 nA). Cs-based solution suppressed potassium channel activity to improve voltage-clamp recordings. While Cs likely also altered the resting potential and amplitude of the recorded membrane voltage response, this was not expected to substantially alter the timing of the stimulus evoked response. *Post hoc* assessment of dendritic morphology from dye fills was used to confirm α-type identity of all electrophysiologically recorded ganglion cells. To test for inhibitory circuit contributions to phase advancing in ganglion cells we used a loss-of-function approach. Glycine receptors were blocked with strychnine (1 μm; Tocris); GABA_a_ receptors were blocked with SR95531 (gabazine, 50 μm; Tocris); GABA_a-_ρ (former GABA_c_) receptors were blocked with the selective, competitive antagonist TPMPA (50 μm; Tocris).

### Visual stimuli

Visual stimuli were generated in MATLAB (MathWorks) using the Psychophysics toolbox (Brainard, 1997) version 3.0.14 for Mac OSX. The stimulus set used throughout consisted of a leftward moving, rightward moving and stationary flashed spot of either +100% (light increment) or −100% (light decrement) Michelson contrast presented on a wide-field (3.0 × 4.0 mm) mid-level gray background in pseudo-random order, to stimulate both the ON and OFF pathway of the retina, respectively. Unless stated otherwise, the spot diameter was 220 μm and the spot moved at a constant velocity of 1340 μm/s (32°/s); the stationary flashed spot was presented for five video frames (∼80 ms) centered in the imaging window. This duration was chosen because it was the shortest duration that evoked a maximal response amplitude. Stimuli were focused onto the photoreceptor layer using a customized DLP video projector (HP AX325AA; Hewlett-Packard) fitted with a UV LED (λ_max_ = 395 nm after the projection optics).

### Data analysis

We used custom-developed software (FluoAnalyzer; B. G. Borghuis) to quantify stimulus-evoked fluorescence responses. For calcium imaging with GCaMP6f in the ganglion cell layer, regions of interest (ROIs) following the perimeter of the soma for each cell were hand-drawn (Fig. 2*A*,*B*). For iGluSnFR analysis in the IPL, we computed the fluorescence response averaged across the entire imaged area. Because dark regions between labeled structures contribute negligibly to the baseline fluorescence, averaging across the entire field of view gives similar results to when ROIs are defined to include fluorescent structures only (Borghuis et al., 2011).

Responses were measured as fluorescence change from baseline fluorescence; ΔF/F_0_. Phase advancing was measured by comparing the response onset time (osPA) and response time-to-peak (ttpPA) for the moving versus flashed spot stimulus. The rationale for evaluating phase advancing of response onset is that for initiating a behavioral response, the timing of neuronal response onset is critical. Furthermore, it has been demonstrated that in dynamic visual encoding the very first spikes fired by a neuronal population carry the most information (Gollisch and Meister, 2008). Response time-to-peak, on the other hand, is similarly relevant as it drives the center of mass of the ganglion cell population response that was central to the demonstration of predictive coding in previous work (Leonardo and Meister, 2013). To evaluate response timing as accurately as possible, we corrected for horizontal spatial offset of each cell’s receptive field within the imaged area. This correction was necessary because while the (stationary) flashed stimulus arrives at all cells in the imaged area simultaneously, the moving stimulus reaches each ganglion cell within an imaged population at slightly different time points depending on the cell’s receptive field location with respect to the center of the imaged area, resulting in overestimates or underestimates of the response time for the moving stimulus.

**Figure 1.**
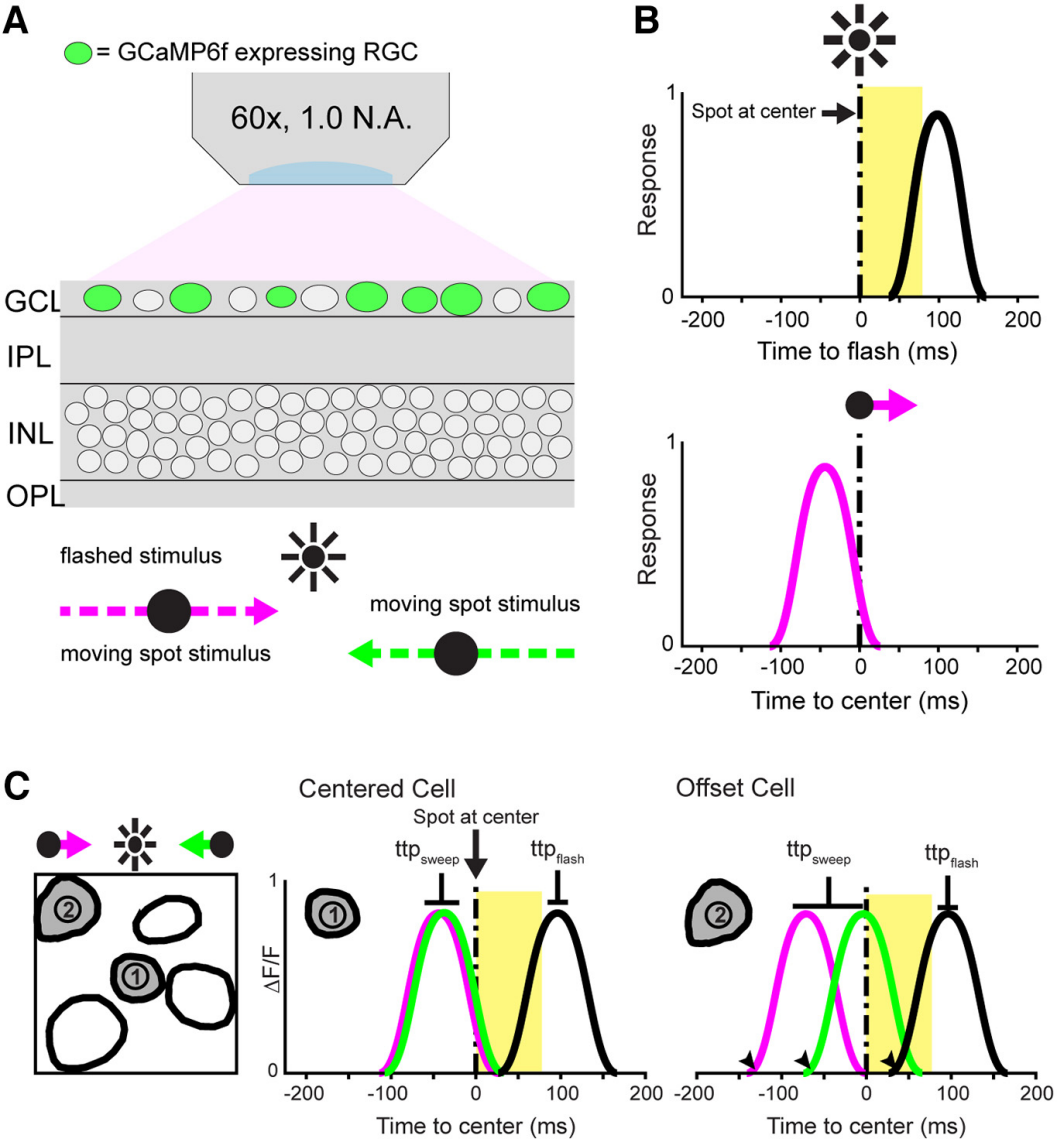
Imaging object motion-evoked ganglion cell responses. ***A***, Schematic cross-section of the whole-mount Thy1-GCaMP6f mouse retina preparation. Calcium responses of GCaMP6f-expressing ganglion cells (green) were measured using two-photon fluorescence imaging (N.A., numerical aperture). Visual stimuli comprised a moving or flashed spot of positive or negative contrast (light or dark spot; 100% Michelson contrast; 160 μm in diameter) on a mid-level gray background, focused onto the photoreceptors using the microscope’s condenser lens. The illuminated area on the retina was 4.5 × 3.5 mm. ***B***, Schematic ganglion cell calcium response to a flashed spot (timing indicated in yellow) with expected visual response latency (top), and to a moving spot that crossed the center of the imaged area at *t* = 0 s (t_0_). The moving spot in this schematic causes a phase-advanced response onset and response time-to-peak (bottom). ***C***, Schematic demonstrating the impact of spatial location within the imaged retinal area, and the mathematical operation to correct for it (for details, see Materials and Methods). The impact of receptive field spatial offset with respect to the center of the recorded area (compare cell 1 vs cell 2) is corrected by averaging the response onset and time-to-peak values for the rightward (magenta) and leftward (green) motion directions (for details, see Materials and Methods). Because spatial offset within the recorded area is small (<±21 μm), its impact on the response to the flashed spot stimulus is negligible.

For example, a rightward-moving stimulus would reach a cell with receptive field on the left side of the imaged area earlier compared with a cell with receptive field on the right side of the imaged area. Obviously, it would be incorrect to interpret the earlier response of the left-side cells as more strongly phase advancing. Thus, we corrected for the relative position of the receptive fields of all cells within the recorded area by presenting motion stimuli in two directions, rightward and leftward (Fig. 1). We then calculated for each stimulus direction the onset/peak response time and averaged these measured response times to obtain the reported onset/peak response time value for each individual cell. This correction does not make assumptions about RF shape symmetry, uniformity, or response dynamics, and provides a first-order adjustment for horizontal spatial offset: for a cell with RF offset to the left, the rightward moving stimulus arrives at the receptive field earlier by the same amount that it comes later for the leftward stimulus. Averaging the two measured response time values gives the values one would expect if the receptive field were centered in the imaged field of view. Note that this approach does not address specific receptive field nonlinearity, such as in direction selective cells, which may not respond to the nonpreferred motion direction. In these cases, some effect of spatial position within the imaged area on measured timing remains.

Using the averaged response time measures (response onset and response time-to-peak) we calculated the phase advancing (*PA*) value by subtracting the average time of the response to the moving spot (*tr_sweep_*) from the time of the response to the flashed spot (*tr_flash_*):

(1) $$PA=tr_{flash}-\bar{tr_{sweep}}.$$

Response onset time was measured as the time point when the response was 3 SDs above baseline. Baseline was obtained from 500 ms of recording with gray background exposure immediately preceding each stimulus presentation. While we could readily measure the response time-to-peak for each recorded cell, in some cells relatively large baseline variability combined with a low response amplitude precluded reliable measurement of the response onset time. Therefore, in the presented data, sample size (*n*) for response onset time is typically smaller than the sample size for response time-to-peak. Cells with a *PA* value >5 ms were classified as phase advancing. The 5-ms criterion was chosen to include cells with a motion versus flash-evoked response time that was shorter on a time scale that is biologically relevant and sufficiently robust against trial-to-trial response variability.

In the glutamate imaging experiments, quantal synaptic vesicle release is integrated by the iGluSnFR sensor protein in space (1- to 2-μm point spread function) and in time (8 ms rise time constant, 35 ms decay time constant), and observed as a graded (continuous) function in time. Fluorescence images were obtained at a frame rate of 15 fps (67-ms frame interval). To obtain an accurate measure of the onset/peak response time, the measured fluorescent response was fit by cubic spline interpolation and response onset times were obtained from the response fit.

Statistical analysis was performed in MATLAB, Microsoft Excel, or GraphPad Prism. Results are presented as mean ± SEM throughout. Statistical significance was determined using a paired or unpaired *t* test for comparison between two groups. For data with an apparent non-normal statistical distribution, we used the Mann–Whitney *U* test. For multigroup comparison, significance was determined using a one-way ANOVA and Tukey’s *post hoc* test. A *p* value < 0.05 was considered statistically significant.

### Spatiotemporal filter and model simulations

We used reverse correlation to measure the spatial and temporal receptive fields for the recorded ganglion cells (Sakai, 1992; Chichilnisky, 2001). To collect ganglion cell spatiotemporal filters, a ∼700 × 700 μm white noise checkerboard stimulus with randomly flickering ∼22 × 22 μm patches of either +100% or −100% contrasts were presented at a refresh rate of two monitor frames (30 stimulus fps) for 5000 stimulus frames (∼3 min). Reverse correlation analysis was performed using custom MATLAB algorithms to obtain the ganglion cell’s spatiotemporal filter characteristics.

To model ganglion cell excitatory and inhibitory synaptic input and membrane voltage responses, we convolved the stimulus with the ganglion cell’s spatiotemporal filter measured in voltage clamp at the reversal potential for chloride (excitation; −60 mV) and cations (inhibition; 0 mV) or in current clamp (membrane voltage response; I = 0). This gives the linear prediction of the ganglion cell’s response to the flashed or moving stimulus. For comparison, amplitudes of the measured and modeled responses were normalized using Equation 2:

(2) $$x_{norm}=\frac{x-\bar{x}}{\sigma },$$

where *x* is the response, $\bar{x}$ is the mean of the response and σ is the SD. Phase advancing was measured from the normalized model responses as osPA and ttpPA as described for the neuronal responses.

## Results

### GCaMP6f expression in the mammalian retina

**Figure 2.**
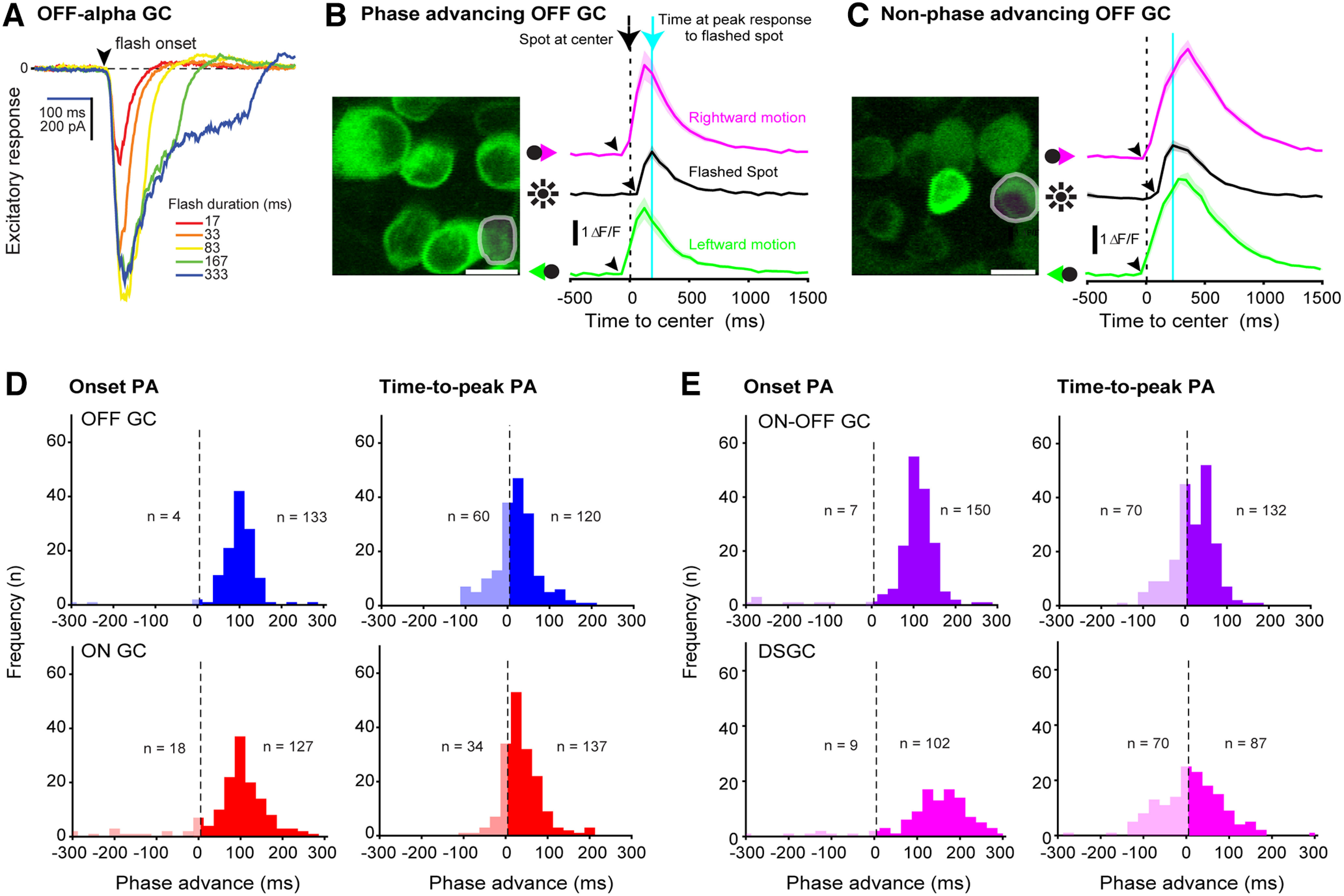
Optical recordings show phase-advanced responses in multiple ganglion cell populations. ***A***, We made electrophysiological whole-cell recordings of excitatory current responses to flashed contrast spots to determine the flash duration that gave the maximum response amplitude without prolonging the response. We used this flash duration (5 monitor frames; 83 ms) throughout this study. ***B***, Left, Two-photon fluorescence image of GCaMP6f-expressing cells in the ganglion cell layer. Right, Calcium responses of an example phase advancing OFF-type ganglion cell in the imaged population (gray circle in left panel; ttpPA = 56 ms, osPA = 119 ms). The vertical dashed black line indicates t_0_, i.e., when the spot crossed the center of the imaging window. Arrowheads indicate the measured response onset time; the vertical cyan line shows the peak response time for the flashed spot. Shaded area represents ±SEM; scale bar = 10 μm. ***C***, Example OFF-type ganglion cell with ttpPA = −104 ms considered not phase advanced, and osPA = 100 ms considered phase advanced. ***D***, ***E***, Population data histograms for all identified cell populations (OFF, ON, ON-OFF non-DS, and DS) showing response onset time (left) and response time-to-peak (right). Shaded columns (left) show cells classified as not phase advancing (<5 ms difference between response to moving vs flashed spot). Representative examples of phase advancing and nonphase advancing ON GC, ON-OFF GC, and DSGC populations are shown in Extended Data Figure 2-1. Scatter plots of response onset phase advancing versus response time-to-peak phase advancing for the recorded cell populations are shown in Extended Data Figure 2-2. A table in Extended Data Figure 2-3 summarizes the measurements of phase advancing for each group.

We measured visual motion-evoked responses in retinal explants of GCaMP6f transgenic mice (Chen et al., 2013). GCaMP6f expression under control of the *Thy1* promoter stochastically labels neurons with differences in expression pattern between founder lines (Dana et al., 2014). We selected three founder lines (GP5.5, GP5.11, and GP5.17) based on reported expression patterns (Dana et al., 2014) and assessed GCaMP6f expression and visually-evoked responses using two-photon fluorescence imaging. GP5.5 did not show GCaMP6f expression in the retina (data not shown). Robust GCaMP6f expression was observed in retinal ganglion cells in both the GP5.11 and GP5.17 lines (Fig. 2*A*), and both lines were used in experiments. Because expression patterns were similar, they were used interchangeably and data obtained from the two lines were combined throughout this study.

10.1523/ENEURO.0270-22.2022.f2-1 Extended Data Figure 2-1 Optical recordings show phase-advanced responses in diverse ganglion cell populations. Two-photon fluorescence images of GCaMP6f-expressing cells in the ganglion cell layer (left) and accompanying GCaMP6f fluorescence responses for the indicated cell (gray circle). Scale bar = 10 μm. The vertical dashed black line indicates t_0_, i.e., when the spot crossed the center of the imaging window; vertical cyan line shows the peak response time for the flashed spot. We measured phase-advanced responses in all functionally defined ganglion cell populations shown here (ON, ON-OFF non-DS, and DS). Download Figure 2-1, TIF file.

10.1523/ENEURO.0270-22.2022.f2-2 Extended Data Figure 2-2 Scatter plots of response onset versus response time-to-peak phase advancing for all individual cells identified in the GCaMP6f population imaging experiments (ON GCs: *n* = 171; OFF GCs: *n* = 180; DSGCs: *n* = 157; ON-OFF GCs: *n* = 202). Download Figure 2-2, TIF file.

**Figure 3.**
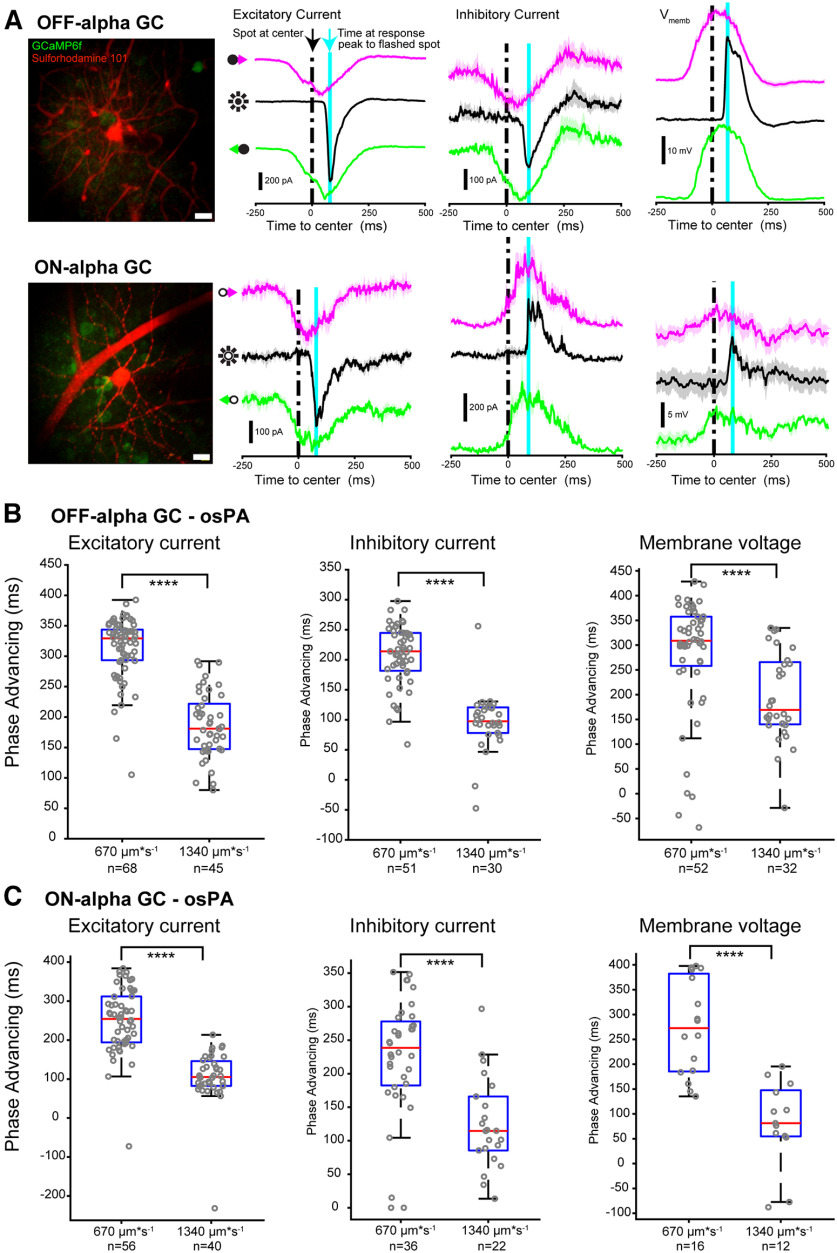
OFF-α-type and ON-α-type ganglion cells receive phase-advanced excitatory and inhibitory synaptic input. ***A***, Two-photon fluorescence image of an OFF-α ganglion cell (top) and ON-α ganglion cell (bottom) filled with the red fluorophore Sulforhodamine 101 during targeted electrophysiological whole-cell recording (scale bar = 20 μm). Traces (right) show the cells’ respective excitatory current (V_hold_ = −60 mV), inhibitory current (V_hold_ = 15 mV), and membrane voltage response to a rightward (magenta line) and leftward (green line) moving spot, and a stationary flashed spot (black line). Spot velocity = 1340 μm s^−1^; shaded area, ± SEM. ***B***, Phase advancing of response onset time for the recorded OFF-α-type ganglion cell population. Measurements included spots moving at two speeds (670 vs 1340 μm s^−1^). Box and whisker plot, median (red); 25th and 75th percentiles (top and bottom edges, blue); error bars, ±1 SD. Gray circles represent data from individual cells. Statistical comparison: Mann–Whitney *U*, all ****p < 0.0001 (left: *U* = 149, *n* = 68, 45; center: *U* = 102, *n* = 51, 30; right: *U* = 393, *n* = 52, 32). ***C***, Same as ***B***, for all recorded ON-α-type ganglion cells. All ****p < 0.0001 (left: *U* = 114, *n* = 56, 40; center: *U* = 140, *n* = 36, 22; right: *U* = 12, *n* = 16, 13). Response time-to-peak data for the recorded population is shown in Extended Data Figure 3-1.

10.1523/ENEURO.0270-22.2022.f2-3 Extended Data Figure 2-3 Values for response onset and time-to-peak phase advancing cells obtained from GCaMP6f imaging data. Download Figure 2-3, PS file.

We recorded visually-evoked calcium responses of ganglion cell populations (5–15 cells per imaged area) in forty retinas from 23 animals. Each responsive cell in the dataset was classified as OFF, ON, or ON-OFF based on the response to dark and light flashed spots. We then assessed for cells in each class the presence and prevalence of phase-advanced response timing. To this end, we recorded the cells’ responses to moving spots and to flashed stationary spots, and compared the time to response onset and time to response peak for these two stimuli.

### OFF-type and ON-type ganglion cells phase advance

Our data set included 180 OFF-type ganglion cells and 171 ON-type ganglion cells. We found that the majority of OFF-type ganglion cells have phase-advanced responses. The response traces for a phase advancing OFF type ganglion cell example cell in Figure 2*B* illustrate how the visually evoked fluorescence response to a moving spot starts (arrowhead) before the stimulus is at the receptive field center (dashed vertical line), and peaks before the response to the flashed spot. Of the 180 responsive OFF cells, we could reliably determine the response onset time for 137 cells (see Materials and Methods). Of these cells, 133 (97%) had a response onset time that preceded the response to a flashed stationary spot. Thus, a phase-advanced response onset was ubiquitous in the recorded population. The mean onset phase advance value (osPA), which measures how much the response onset for a moving spot precedes that for a flashed spot, was 103.2 ± 2.9 ms. Of the 180 responsive OFF cells, 120 (67%) had a phase-advanced response based on their response time-to-peak (mean ttpPA 46.7 ± 3.8 ms). Response time-to-peak of the remaining 60 cells (33%) lagged that of the flashed spot (mean ttpPA −32.5 ± 4.3 ms). Because nearly all cells exhibited a phase-advanced response onset for the motion stimulus, the difference between cell populations with a phase advanced versus phase delayed time-to-peak must reflect differences in the cells’ temporal integration.

The above phase advance values were obtained using the center of the spot as the spot’s current position. The rationale for measuring timing with respect to object center location is that for interacting with a moving object, the object’s center is a key biological variable. For example, in prey capture, the center of mass of a prey item is arguably most important (Borghuis and Leonardo, 2015). To measure, instead, the timing of the fluorescence response with respect to the position of the leading edge of the moving spot, we subtracted the temporal difference between the spot’s edge and its center, 83.3 ms (spot diameter of 220 μm, velocity 1340 μm s^−1^). When we compared the timing of the flashed response to the timing of the stimulus leading edge crossing the center of the imaged area, 69% of cells still showed a phase-advanced response onset (mean osPA = 34 ms), and 8% of cells still showed a phase-advanced response time to peak (mean ttpPA = 44 ms).

Our data set included 171 functionally identified ON-type ganglion cells and their response timing was assessed with a moving or flashed light spot against a gray background (100% Michelson contrast. Response onset time could be determined for 145 cells. Of these, 127 cells (88%) showed a phase-advanced response onset (mean osPA = 111.6 ± 4.3 ms) and 137 cells (77%) showed a phase-advanced response time-to-peak (mean ttpPA = 51 ± 4.7 ms; Fig. 2*C*). The response peak of the remaining 34 cells lagged behind that of the response to the flashed spot.

Correcting for the stimulus leading edge using the edge time offset, 61% of the cells had a phase-advanced response onset (mean osPA = 50 ms) and 11% of cells had a phase-advanced response time-to-peak (mean ttpPA = 47 ms). These results show that like their OFF-type ganglion cell counterparts, the response to object motion of mouse ON-type retinal ganglion cells is phase advanced.

### Additional phase advancing ganglion cell types

We identified two additional populations of ganglion cells in the fluorescence calcium imaging data set: ON-OFF direction-selective ganglion cells (DSGCs) and ON-OFF non-DSGCs. Because our horizontally moving stimulus (all combinations of leftward moving, rightward moving, light spot, and dark spot) only identified DSGCs tuned to horizontal motion directions, the population of cells classified as ON-OFF non-DSGC likely included ON-OFF DSGCs tuned to nonhorizontal motion directions, i.e., ventral to dorsal, or dorsal to ventral. Phase advancing in such “vertically” tuned ON-OFF DSGCs based on response onset (first-spike) using moving bars has been reported previously in Hb9 positive DSGCs (Trenholm et al., 2013a).

Out of 157 horizontally tuned DSGCs, 87 (55%) showed a phase-advanced response, with a mean ttpPA value of 70.9 ± 7.2 ms. Of the 111 DSGCs for which the response onset time was defined, 102 (92%) had a mean osPA value of 172.4 ± 7.4 ms. (Fig. 2*C*). Correcting for the leading edge using the edge time offset showed an osPA of 101 ms in 83% of DSGCs and a ttpPA of 48 ms in 7% of DSGCs.

In the ON-OFF non-DSGC population, response onset could be determined for 157 ON-OFF cells. Of these 150 (96%) showed a phase-advanced response (mean osPA value of 116.5 ± 3.1 ms). Correcting for the leading edge of the moving spot using the edge time offset, the osPA was 44 ms in 78% of cells and ttpPA was 34 ms in 4% of ON-OFF ganglion cells. Based on response time-to-peak, 132 out of 202 cells (65%) had a phase-advanced response (mean ttpPA: 47.9 ± 3.9 ms; Fig. 2*C*).

### Excitatory and inhibitory synaptic inputs to OFF-α ganglion cells phase advance

Earlier work proposed at least two different mechanisms for phase advancing: cell intrinsic spike adaptation based on recordings in salamander retina (Leonardo and Meister, 2013) and circuit specific inhibition, and not excitation, in goldfish retina (Johnston and Lagnado, 2015). To determine the origin of the phase-advanced response in mouse retinal ganglion cells we studied phase advancing at the level of synaptic currents using whole-cell voltage-clamp recording. First, we recorded excitatory synaptic inputs to OFF-α-type ganglion cells using dark spots (∼220 μm in diameter) on a gray background, as in the calcium imaging experiments. The flashed spot (∼80 ms in duration) evoked a large inward current (400–600 pA; Fig. 3*A*, left), demonstrating strong transient excitation. The principal origin of this current is glutamate released from presynaptic bipolar cells. The moving dark spot similarly evoked an inward current, but this current was more sustained, likely because the spot was present over the ganglion cell’s receptive field longer. The OFF-α ganglion cell example shown in Figure 3*A* had osPA and ttpPA values of 228 and 36 ms, respectively. While the flash-evoked response slope was steeper, the motion evoked response still peaked first because of its earlier onset. This example was representative of the recorded population (Extended Data Fig. 3-1).

10.1523/ENEURO.0270-22.2022.f3-1 Extended Data Figure 3-1 A, Response time-to-peak phase advance values of OFF-α ganglion cells measured using electrophysiological whole-cell recordings at the level of the excitatory current (i), inhibitory current (ii), and membrane voltage (iii). The stimulus set comprised spots moving at two velocities. Faster moving spots gave smaller time-to-peak phase advance values. B, As A, for ON-α ganglion cells. Download Figure 3-1, TIF file.

The mean osPA value for all recorded OFF-α ganglion cells was 187 ± 7.9 ms (*n* = 45 cells; velocity 1340 μm s^−1^). The mean ttpPA value was 42 ± 2.4 ms (*n* = 47 cells). Stimulated with a slower moving spot (670 μm s^−1^), phase advancing in OFF-α ganglion cells increased from 187 ± 7.9 to 314 ± 6.2 ms (*n* = 68 cells; *p* < 0.0001; Fig. 3*B*) and ttpPA from 42 ± 2.4 to 78 ± 4.4 ms (*n* = 71 cells; *p* < 0.0001; Fig. 3*B*).

Next, we assessed the response timing of inhibitory synaptic input to ganglion cells. Inhibitory synaptic currents were isolated by whole-cell voltage clamp at the reversal potential for cations corrected for the junction potential, +15 mV. The dark spot stimulus caused a decrease in inhibition (dis-inhibition) in OFF-type ganglion cells (inward current; Fig. 3*A*,*B*, middle). Dis-inhibition is characteristic of OFF-α ganglion cells and is explained by decreased tonic inhibition from amacrine cells (Manookin et al., 2008; Van Wyk et al., 2009). Upon leaving the cell’s receptive field the dark spot caused an increase of inhibitory current (Fig. 3*A*). Stimulated with the faster moving spot (1340 μm s^−1^) the mean osPA of the OFF-α ganglion cells was 96 ± 9.0 ms (*n* = 30 cells) and the mean ttpPA was 48 ± 2.4 ms (*n* = 32 cells). Stimulated with the slower moving spot, osPA and ttpPA values increased (osPA, fast: 96 ± 9 ms; slow: 208 ± 7.1 ms; *p* < 0.0001; ttpPA, fast: 48 ± 2.4 ms, slow: 86 ± 5 ms; *p* < 0.0001; Fig. 3*B*). Thus, we found phase advancing in both excitatory and inhibitory input currents and in both the magnitude scaled negatively with stimulus velocity: greater velocity results in smaller PA, similar to previous work in salamander (Berry et al.,1999).

To determine how the integration of synaptic inputs leads to phase advancing, we recorded the same OFF-α ganglion cells in current clamp mode (I = 0 pA) to measure the membrane voltage response. Sodium channel and potassium channel blockers (2 mm QX-314 and 120 mm cesium methanesulfonate) in the intracellular pipette solution eliminated action potentials and voltage gated potassium conductances. The example OFF-α ganglion cell (Fig. 3*A*, right) at the level of the membrane voltage response showed a ttpPA of 15 ms and osPA of 155 ms. The osPA values recorded at the faster speed were significantly smaller compared with the slower speed (fast: 197 ± 15.9 ms vs slow: 282 ± 16.4 ms; *p* = 0.0008; Fig. 3*B*) and the same was observed for the ttpPA values (fast: 51 ± 2.5 ms vs slow: 86 ± 4.2 ms; *p* < 0.0001; Fig. 3*B*, left).

These data show that OFF-α ganglion cells in mouse have phase-advanced responses similar to large soma OFF-type ganglion cells in salamander (Berry et al., 1999; Leonardo and Meister, 2013). Next, we tested whether this feature extends also to ON-α ganglion cell synaptic currents, which were not reported on in previous studies.

### Excitatory and inhibitory synaptic inputs to ON-α ganglion cells phase advance

ON-α ganglion cells receive synaptic input from receptive field subunits that are more strongly nonlinear than those serving OFF-α ganglion cells (Schwartz et al., 2012). These subunits signal local contrast changes and rectify the light input, resulting in increased sensitivity to fine spatial features in ON-α cells (Demb et al., 1999; Turner et al., 2018). It is not known whether increased nonlinear receptive field properties of ON-α ganglion cells contribute to phase-advanced signaling. The following experiments were designed to address this.

Recorded during voltage clamp at the reversal potential for chloride, a flashed light spot evoked in ON-α cells a typical excitatory (inward) current of 150–250 pA (Fig. 3*A*). Across the recorded ON-α ganglion cell population, the slower moving spot caused a more phase-advanced response compared with the faster spot, similar to the OFF-α ganglion cells, above (osPA, fast: 105 ± 10.7 ms vs slow: 249 ± 11 ms; *p* < 0.0001; ttpPA, fast: 49 ± 5 ms vs slow: 99 ± 4.8 ms; *p* < 0.0001; Fig. 3*C*).

Next, we measured response timing of the inhibitory synaptic input to ON-α ganglion cells. Stimulation with a light spot in ON-α ganglion cells evoked a fast transient increase in inhibitory current that was phase advanced (Fig. 3*A*, middle). As in the excitatory synaptic current, the response to the slower moving spot was more phase advanced than the response to the faster moving spot (osPA, slow: 225 ± 14.9 ms, *n* = 36 cells vs fast: 125 ± 14.8 ms, *n* = 22 cells; *p* < 0.0001; ttpPA, fast: 39 ± 5.8 ms, *n* = 39 cells vs slow: 74 ± 8.5 ms, *n* = 39 cells; *p* < 0.0001; Fig. 3*C*).

When examined at the level of the membrane voltage response, ON-α ganglion cell responses were phase advanced (example cell shown in Fig. 3*A*, bottom, right; osPA: 119 ms, ttpPA: 68 ms), and here, too, there was a significant difference between the phase advancing values when stimulating with faster compared with slower moving spots (osPA, slow: 274 ± 24.1 ms vs fast: 76 ± 25.4 ms; *p* < 0.0001; ttpPA, slow: 150 ± 11.2 ms vs fast: 82 ± 9.24 ms; *p* = 0.0003; Fig. 3*C*).

These data, obtained with targeted whole-cell recordings, demonstrate that object motion-evoked excitatory and inhibitory synaptic input in both OFF-α-type and ON-α-type ganglion cells is phase advanced.

### A linear-nonlinear (LN) model of phase advancing in α-type ganglion cells

The dependence of the magnitude of phase advancing on stimulus speed can be a direct result from the receptive field spatial extent. If this is true, then this should be captured accurately by a simple spatiotemporal receptive field model. A powerful model for assessing and predicting the visual responses of retinal ganglion cells has been the LN model (Chichilnisky, 2001). The LN model comprises a linear spatiotemporal weighing function to represent the ganglion cell receptive field and a static-nonlinear transfer function that describes the relationship between the linearly integrated input and the cell’s output. The static-nonlinearity is a function that fits the modeled data to the measured responses with the smallest root mean square error (Chichilnisky, 2001). The LN model has been demonstrated to predict retinal responses to a wide range of stimuli and stimulus conditions (Berry et al., 1999; Schwartz et al., 2007; Leonardo and Meister, 2013). However, previous work in salamander showed that it fails to recapitulate the phase-advanced responses measured in this species, and a gain-control feedback loop (LfN), i.e., an added dynamic nonlinearity, was necessary to enable it to do so (Berry et al., 1999; Leonardo and Meister, 2013).

**Figure 4.**
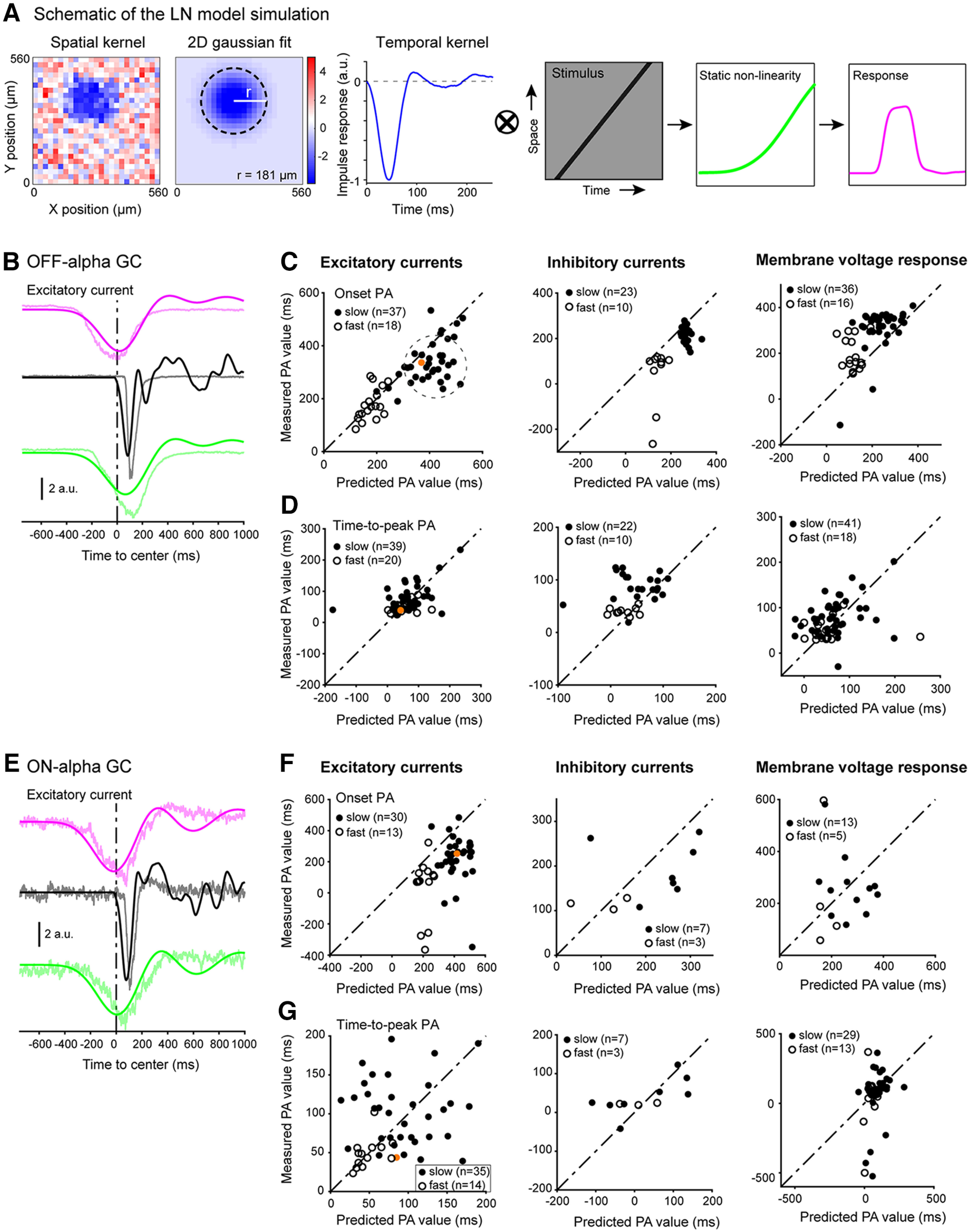
LN receptive field model predicts timing of object motion-evoked responses in α GCs. ***Ai***, Example linear (L) approximations of the spatial receptive field (RF; top, left) and temporal filter characteristic (bottom) of an OFF-α GC, measured using white noise checkerboard stimulation and reverse-correlation analysis. Model simulations used the temporal filter and a 2D difference-of-Gaussians fit to the measured spatial receptive field. ***Aii***, Schematic of the LN model used to predict stimulus-evoked ganglion cell synaptic current and membrane voltage responses. The visual stimulus (left) was convolved with the measured spatiotemporal RF to obtain a linear response prediction. This linear response prediction was scaled nonlinearly (N) for each cell using the static nonlinear transfer function (green) obtained from the measured white noise response. The resulting LN model output (magenta) was compared with the measured response to the moving and flashed spot stimuli. ***B***, LN model response (dark traces) and recorded response (light traces) for an example OFF-α ganglion cell. ***C***, Scatterplot of the measured osPA values versus the modeled values based on excitatory current (left), inhibitory current (center), and membrane voltage response (right). Dashed line, unity; orange point represents the example cell shown in ***B***. ***D***, Same as ***C*** but for ttpPA values. ***E–G***, Same as ***B–D***, for ON-α ganglion cells.

We tested whether the conventional LN model captured the measured response timing of mouse α-type ganglion cells at the level of the excitatory and inhibitory current and the membrane voltage response. To this end we used each cell’s spatiotemporal receptive field and static nonlinearity measured with a white noise checkerboard stimulus during voltage and current clamp. We then convolved the LN model with the flashed and moving spot to compute the response predicted by the LN model (Fig. 4*A*) and compared it to the recorded response to these same stimuli.

Figure 4*B* shows an example of the predicted excitatory synaptic input overlaid on the measured excitatory response of an OFF-α ganglion cell. We obtained data for a population of OFF-α ganglion cells (OFF-α, *n* = 37; ON-α, *n* = 33) and for each cell compared the phase advance values of the measured response with that of the LN model (Fig. 4*C*,*D*; orange dot represents the example data shown in Fig. 4*B*). We found statistically significant differences between the model osPA and the recorded osPAs for the slower moving spot (670 μm s^−1^; dashed circle in Fig. 4*C*, left panel; mean difference of measured – model: excitatory current −63.3 ± 12.7 ms, *n* = 37 pairs, *p* < 0.0001; inhibitory current, −47.8 ± 9.4 ms, *n* = 23 pairs, *p* < 0.0001; membrane voltage, 75.4 ± 14.1, *p* < 0.0001). There was no significant difference in the onset time of the excitatory response to faster moving spots, nor in the response time-to-peak for slow or fast moving spots. Our interpretation is that the slow moving spot activates a nonlinear mechanism not captured in the model, possibly contrast gain control.

Next, we performed the same comparison for ON-α ganglion cells (example shown in Fig. 4*E*; population data, Fig. 4*F*,*G*). Here, the only statistically significant difference in phase advance values between the measured and modeled data were that for the slow spot osPA values of the modeled excitatory currents overestimated the amount of phase advancing (mean difference of measured – model: excitatory current, −193.6 ± 30.3 ms, *n* = 33 pairs, *p* < 0.0001; inhibitory current, −45.8 ± 39.7 ms, *n* = 7 pairs, *p* = 0.293 n.s.; membrane voltage, −34.5 ± 54.1, *p* = 0.539).

Our computational analysis shows that the LN model largely recapitulates the timing of response time-to-peak of OFF-α and ON-α cells, but in both cell types overestimates phase advancing of onset of excitation as well as inhibition for slow moving spots. The model underestimates phase advancing of the membrane voltage response also for slow moving spots. Discrepancy between the model prediction and recorded response could be explained by a dynamic nonlinearity, possibly contrast adaptation, that is not captured by the LN model.

### Phase advancing in ganglion cells does not require GABA_A_-ergic inhibition

**Figure 5.**
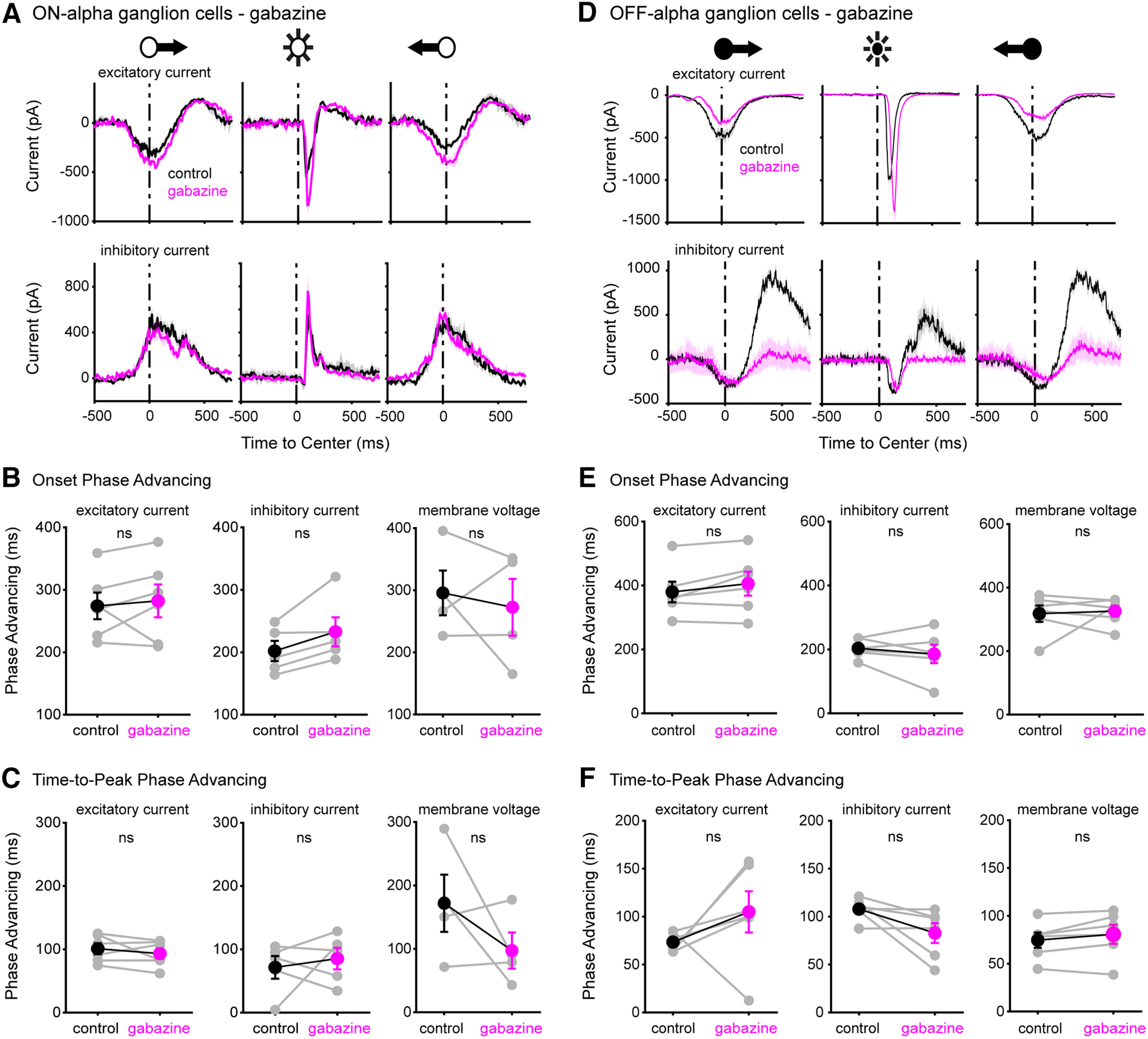
Phase advancing in ON-α and OFF-α GCs does not require GABA_a-_ergic inhibitory signaling. ***A***, Excitatory (top) and inhibitory (bottom) currents obtained with electrophysiological whole-cell recordings from an example ON-α ganglion cell to moving and flashed spot stimuli under control conditions (black) and in the presence of gabazine (magenta). ***B***, ***C***, Response onset and time-to-peak for the recorded ON-α GC population under control conditions (black) and in the presence of gabazine (magenta). ***D–F***, As ***A–C***, for OFF-α GCs. Extended data figures show results obtained using the pharmacological blockers TPMPA (Extended Data Fig. 5-1) and strychnine (Extended Data Fig. 5-2).

Electrophysiological whole-cell recordings (Fig. 3) show that the excitatory and inhibitory synaptic currents in ON-α and OFF-α ganglion cells is phase advanced. We asked whether the phase-advanced response relies on inhibitory circuit interactions, as has been demonstrated in goldfish (Johnston and Lagnado, 2015). To answer this we measured response onset and response time to peak of ON-α and OFF-α ganglion cells during whole-cell recordings under control conditions and in the presence of one of the following selective inhibitory neurotransmitter receptor blockers: gabazine (SR-95531), TPMPA, or strychnine, to block GABA_A_, GABA_A_-ρ (formerly known as GABA_C_), and glycine receptors, respectively. While inhibitory receptor block impacted the cells’ current response amplitude, our data show no significant effect on the timing of response onset or response time-to-peak (Fig. 5, Extended Data Figures 5-1, 5-2). Specifically, gabazine increased the amplitude of the stimulus-evoked excitatory current in ON-α cells, and reduced this amplitude in OFF-α cells; the amplitude of the inhibitory response in ON-α cells was unchanged, whereas in OFF-α cells it was strongly reduced (Fig. 5*A*,*D*). TPMPA caused a slight decrease (<15% change) in excitatory and inhibitory response amplitude in ON-α cells, and an increase of similar magnitude of these currents in OFF-α cells (Fig. S4A,D). Strychnine increased the ON-α cell excitatory response and decreased the OFF-α cell excitatory as well as inhibitory response (Fig. S5A,D). The phase advance values measured for the recorded cell population (*n* = 6–7 for each drug condition) based on response onset and response time-to-peak of excitatory current, inhibitory current, and the membrane voltage response remained unchanged following selective pharmacological block of inhibition (Fig. 5*B*,*C*,*E*,*F*; Fig. S4B,C,E,F; Fig S5B,C,E,F).

10.1523/ENEURO.0270-22.2022.f5-1 Extended Data Figure 5-1 Pharmacological block of GABAa-rho receptors does not alter response timing in α-type ganglion cells. ***A***, Excitatory (top) and inhibitory (bottom) currents obtained using electrophysiological whole-cell recording from an ON-α ganglion cell under control conditions (black) and in the presence of selective GABA_a-rho_ (former GABA_c_) receptor blocker TPMPA (50 μm; magenta). ***B***, Response onset phase advancing for the recorded ON-α ganglion cell population (*n* = 6). ***C***, Response time-to-peak phase advancing for the recorded ON-α ganglion cell population. ***D–F***, as ***A–C***, for OFF-α ganglion cells (*n* = 5). Download Figure 5-1, TIF file.

10.1523/ENEURO.0270-22.2022.f5-2 Extended Data Figure 5-2 Pharmacological block of glycine receptors does not alter response timing in α-type ganglion cells. A, Excitatory (top) and inhibitory (bottom) currents obtained using electrophysiological whole-cell recording from an ON-α ganglion cell under control conditions (black) and in the presence of glycine receptor blocker strychnine (1 μm; magenta). B, Response onset phase advancing for the recorded ON-α ganglion cell population (*n* = 6). ***C***, Response time-to-peak phase advancing for the recorded ON-α ganglion cell population. ***D–F***, As ***A–C***, for OFF-α ganglion cells (*n* = 5). Download Figure 5-2, TIF file.

### Glutamate release from bipolar cells is phase advanced

While our electrophysiological whole-cell recordings from α ganglion cells showed that the excitatory synaptic response of these cells is phase advanced (Fig. 3), this does not necessarily mean that the bipolar cell glutamatergic response is phase advanced. Since the excitatory current recorded at the ganglion cell soma reflects excitation integrated across the dendritic arbor, the early onset of the excitatory response to a moving spot can simply be driven by activation of bipolar cells at the ganglion cell’s receptive field perimeter. On the other hand, bipolar cells should be capable of phase advancing simply based on their receptive field diameter. Phase advancing in the bipolar cells could potentially extend phase advancing in a ganglion cell beyond the perimeter of its dendritic field.

To test for phase advancing in motion-evoked glutamate release from bipolar cells we used two-photon fluorescence glutamate imaging with the sensor protein iGluSnFR (Borghuis et al., 2013; Marvin et al., 2013). iGluSnFR was expressed in the ganglion cells via viral transduction following intraocular injection if an AAV viral vector, and synaptic release from bipolar cells was measured by imaging fluorescence changes at the ganglion cell dendritic arbors in the IPL. The whole-mount retina was stimulated with flashed and moving spots, as above. These measurements allowed us to measure osPA and ttpPA values of bipolar cell synaptic release onto ganglion cell dendrites to determine whether phase advancing is present at the level of the bipolar cell output.

Bipolar cells are most strongly activated by spots with a diameter of ∼150 μm (Borghuis et al., 2013). For our visual stimulus, we used a spot that approximated this size (170 μm in diameter), as well as a larger (280 μm in diameter) spot for comparison with the ganglion cell calcium imaging and electrophysiological recordings.

**Figure 6.**
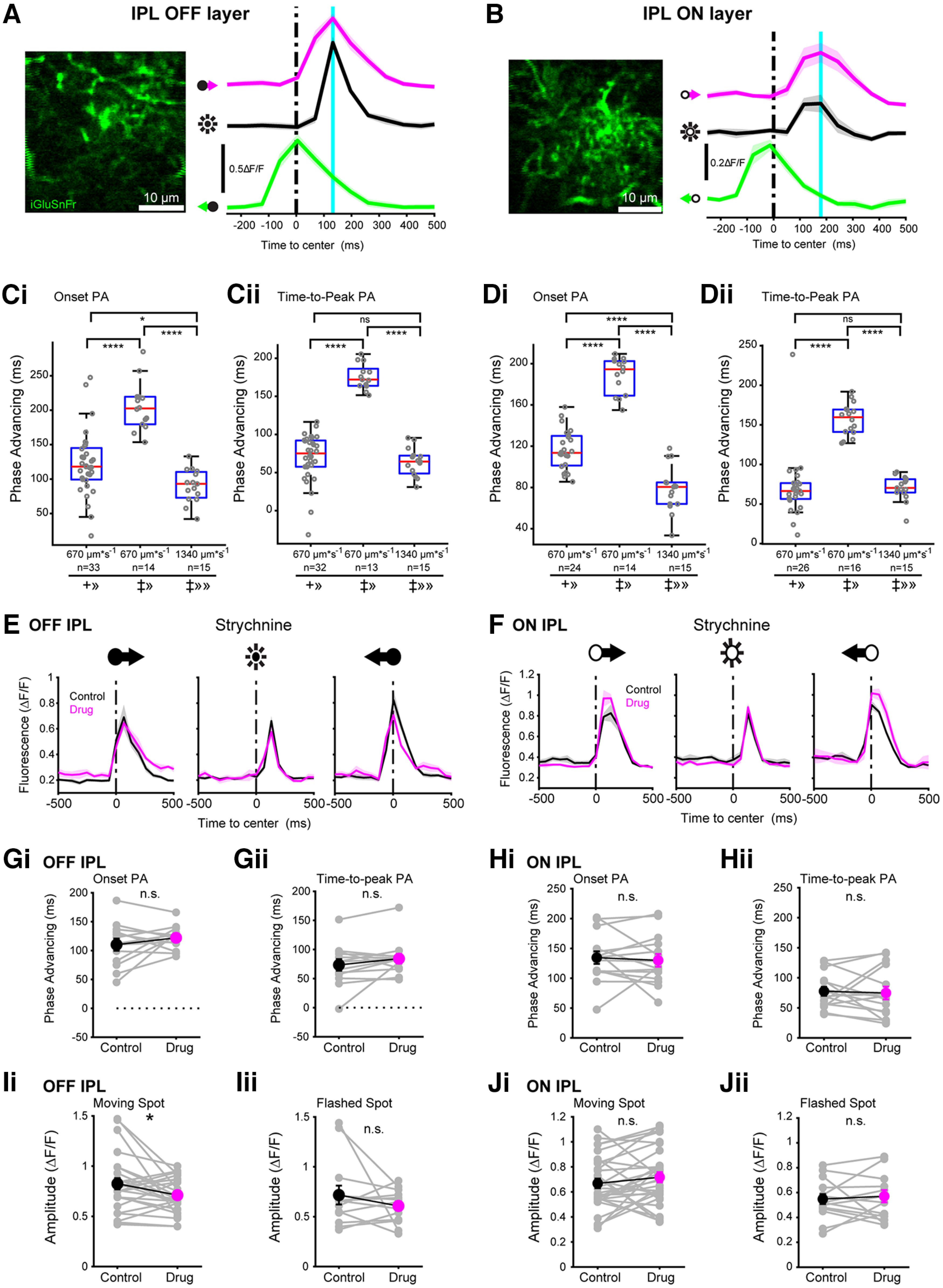
Motion-evoked bipolar cell synaptic glutamate release is phase advanced. ***A***, Left, Two-photon fluorescence image of iGluSnFR-expressing ganglion cell dendrites in the OFF-transient layer of the IPL [imaging depth: ∼32 μm below the ganglion cell layer (GCL); scale bar = 10 μm]. Right, Example iGluSnFR responses to rightward (magenta) and leftward (green) moving spots, and a stationary flashed spot (black line) measured from the labeled processes shown left. The vertical black line indicates when the moving spot crosses the center of the imaging window (t_0_). The vertical cyan line indicates the peak fluorescence response following the flashed spot. Shaded area represents ±SEM across trials (3 repeat minimum). ***B***, As ***A***, for the ON-transient layer of the IPL (imaging depth ∼18 μm below the GCL). ***C***, Box plot of osPA (***Ci***) and ttPA values (***Cii***) from all recorded OFF IPL areas (osPA, *n* = 33; ttpPA, *n* = 32). Red line, median; blue top and bottom lines, 25th and 75th percentiles. Gray circles represent individual recorded IPL areas. Symbols representing size and speed of the moving spots: +, 160 μm spot; ‡, 220 μm; ≫, 670 μm s^−1^; ≫≫, 1340 μm s^−1^. ns, not significant. ns, not significant; *p < 0.05, **p < 0.01, ***p < 0.001, ****p < 0.0001. ***D***, Same as ***C***, for all recorded ON IPL areas (osPA, *n* = 24; ttpPA, *n* = 26). ***E***, Stimulus-evoked iGluSnFR responses in the OFF-transient IPL under control conditions (black) and in the presence of glycine receptor blocker strychnine (magenta). ***F***, As ***E***, for all recordings from the ON-transient IPL. ***Gi***, ***Gii***, Phase advancing values for iGluSnFR responses recorded in the OFF-transient IPL in control compared with drug conditions (osPA, *n* = 13 areas; ttpPA, *n* = 13 areas). Gray lines show individual cells, black and magenta points/error bars show summary mean ± SEM; ns, not significant; *p < 0.05, **p < 0.01, ***p < 0.001, ****p < 0.0001. ***Hi***, ***Hii***, Same as ***G***, for the ON-transient IPL. ***Ii***, ***Iii***, iGluSnFR fluorescence response amplitude in control versus drug conditions (moving spots, *n* = 23 areas; flashed spots, *n* = 15 areas). Gray lines show individual cells; black and magenta points/error bars show summary mean ± SEM. ***Ji***, ***Jii***, Same as ***I***, for the ON-transient IPL.

Figure 6*A* shows an example fluorescence image obtained at a focal plane within the transient OFF layer of the IPL, ∼31 μm below the center of the ganglion cell layer. Using the small spot stimulus, the onset of bipolar cell glutamate release was phase advanced by 126 ms (osPA) and time-to-peak by 72 ms (ttpPA). The average osPA across all imaged areas was 122 ± 8.1 ms (*n* = 33 ROIs) and the average ttpPA was 69 ± 5.6 ms (*n* = 32 ROIs; one area excluded based on insufficient response stability across trials). At this level of the OFF IPL the larger spot (280 μm), moving at 670 μm s^−1^, gave an average osPA of 205 ± 9.3 ms (*n* = 14 ROIs) and an average ttpPA of 176 ± 4.6 ms (*n* = 13 ROIs). The larger spot moving fast (1340 μm s^−1^) gave an average osPA of 90 ± 6.2 ms (*n* = 15 ROIs; Fig. 6*Ci*) and an average ttpPA of 64 ± 4.7 ms (*n* = 15 ROIs; Fig. 6*Cii*), demonstrating robust phase advancing in OFF bipolar cell synaptic release.

Next, we focused our imaging plane at the layer of the ON IPL where the ON-α ganglion cells stratify their dendrites, ∼16 μm below the ganglion cell layer (Fig. 6*B*). The smaller, slow moving light spot (168 μm in diameter; 670 μm s^−1^) at this level of the IPL gave an average osPA of 116 ± 3.9 ms (*n* = 24 ROIs, 14 retinas) and average ttpPA of 70 ± 7.8 ms (*n* = 26 ROIs, 16 retinas). The larger spot (280 μm) moving at the same speed gave an average osPA of 189 ± 4.8 ms (*n* = 14 ROIs) and an average ttpPA of 157 ± 5.1 ms (*n* = 16 ROIs). The larger, faster moving spot (280 μm; 1340 μm s^−1^) gave an average osPA of 78 ± 5.8 ms (*n* = 15 ROIs, 8 retinas; Fig. 6*Di*) and an average ttpPA of 70 ± 4.1 ms (*n* = 15 ROIs, 8 retinas; Fig. 6*Dii*). These glutamate response data show robust phase advancing at the level of the bipolar cell synaptic output.

To test whether the response onset and time-to-peak of the imaged bipolar cell populations depends on interactions with glycinergic inhibitory amacrine cell circuits we selectively blocked glycine receptors through bath-application of strychnine (1 μm) while recording visually-evoked iGluSnFR fluorescence responses from the OFF and ON layers of the IPL. Strychnine did not change phase advancing in the OFF layer compared with control conditions (osPA: *n* = 13, mean difference 12 ± 9.8 ms, *p* = 0.25; ttpPA: *n* = 15, mean difference 10.7 ± 7.0 ms, *p* = 0.15; Fig. 6*Gi*,*Gii*). In the OFF layer of the IPL, strychnine decreased the amplitude of the iGluSnFR response to moving spots but not to flashed spots (flash: *n* = 13, mean difference −0.11 ± 0.09 ΔF/F, *p* = 0.29; moving: *n* = 26, mean difference −0.11 ± 0.05 ΔF/F, *p* = 0.038; Fig. 6*Ii*,*Iii*).

Similar to the OFF layer (above), strychnine did not change phase advancing values in the IPL ON layer compared with control conditions (osPA: *n* = 15, mean difference −5 ± 9.7 ms, *p* = 0.65; ttpPA: *n* = 15, mean difference −2.9 ± 9.6 ms, *p* = 0.77; Fig. 6*Hi*,*Hii*). In contrast to the OFF IPL, strychnine did not affect the iGluSnFR fluorescence response amplitudes in the ON IPL (flash: *n* = 15, mean difference 0.02 ± 0.05 ΔF/F, *p* = 0.63; moving: *n* = 30, mean difference 0.05 ± 0.04 ΔF/F, *p* = 0.23; Fig. 6*Ji*,*Jii*). Response timing in both the OFF and ON IPL was significantly different between the moving and flashed spot stimulus, also after correcting for the spatial offset between the spot center location and the spot’s leading edge.

iGluSnFR imaging showed that bipolar cell glutamate output in both the OFF and ON layers of the IPL is phase advanced. The magnitude of phase advancing is smaller than that of the postsynaptic α-type ganglion cells in accordance with their smaller receptive fields (see Discussion). Our interpretation of these results is that the bipolar cells cause a first, small forward shift in timing of the light-evoked response. This forward shift is subsequently increased in the postsynaptic ganglion cells, by collecting input from bipolar cells across a larger dendritic area.

## Discussion

Accurate timing of neuronal responses is critical for successful interactions with the external world. The goal of this study was to determine to what extent neuronal circuits within the mouse retina help counter response latencies in the visual system, which challenge these interactions. We studied this in the context of the encoding of object motion where fast, accurate encoding is most pertinent.

Using functional imaging and targeted whole-cell electrophysiology in a whole-mount retinal explant preparation, we compared the timing of visual responses evoked by moving versus flashed stationary spots, and assessed response timing with respect to stimulus proximity to the ganglion cell receptive field center. We found that in all major response types (ON, OFF, ON-OFF non-DS, and ON-OFF DS) the onset of the response to a moving spot occurred earlier than the response to a flashed stationary spot. In the majority of the recorded cells the motion-evoked response also peaked earlier than the flash-evoked response but the fraction of cells that showed this was smaller (Fig. 2; Extended Data Fig. 2-1), with 33% of OFF cells and 20% of ON cells showing a delayed time-to-peak for a moving compared with a flashed spot. A forward shift of motion-evoked responses has been described previously in some ganglion cell types in salamander and a particular ganglion cell type (OFF-α) in rabbit (Berry et al., 1999; Leonardo and Meister, 2013), as well as in goldfish (Johnston and Lagnado, 2015). Known as phase advancing, the forward shift in response time demonstrably aids the real-time localization of a moving object (Leonardo and Meister, 2013). Our comprehensive measurements of response timing at the level of the ganglion cell population using calcium imaging, ganglion cell synaptic currents, and presynaptic bipolar cell glutamate release showed that most assayed cell types in mouse show some degree of phase advancing. Phase advancing of response onset scaled with the respective cells’ spatial receptive field extent: it was smallest in bipolar cells and largest for α-type ganglion cells, consistent with work in goldfish, where cells with the smallest receptive fields were also reported to have the least phase-advanced response (Johnston and Lagnado, 2015). While nearly all recorded cells showed a phase-advanced response onset, in a fraction of cells within each identified type (ON, OFF, ON-OFF, and DSGC) response time-to-peak showed a phase delay, indicating that the motion-evoked response in these cells was prolonged, causing increased calcium accumulation for a longer period of time compared with the flash evoked response. How this apparent sustained motion-evoked response contributes to downstream visual processing remains unclear.

Our data show phase-advanced responses in OFF-type ganglion cells including fast (transient) OFF-α-type ganglion cells consistent with previous studies in the salamander and rabbit retina (Berry et al., 1999; Schwartz et al., 2007; Leonardo and Meister, 2013). We additionally show that the motion-evoked response of ON-type ganglion cells, including ON-α cells, as well as ON-OFF-type cells including ON-OFF direction selective cells in mouse are phase advanced.

### Generating a phase-advanced response

Latency of the initial visual response in the outer retina is caused by the response time of the molecular machinery for photo-transduction and synaptic transmission, and cannot be undone. Therefore, any mechanism designed to counter it necessarily relies on prediction (Baylor et al., 1984, 1987). At the single cell level, prediction is first implemented by the physical extent of a neuron’s visual receptive field. If one considers a ganglion cell a pixel-type encoder dedicated to cover a particular location in visual space, then for an apparent “general-purpose” type ganglion cell such as the α cell (large pixel) or the UD/UHD cells (small pixel; Jacoby and Schwartz, 2017) action potential output to a recipient visual area will, on average, correlate most strongly with the presence of a light stimulus located at the receptive field center. Indeed, the statistically “best guess” of a retino-recipient area receiving an action potential from this type of retinal ganglion cell is that there is a stimulus located at the center of that ganglion cell’s receptive field.

The predictive value of a ganglion cell’s action potential output with regards to the presence of a stimulus at a particular location on the retina (and object at the corresponding location in visual space) is established during development and is reflected in the approximately Gaussian spatial weighting function of the cell’s receptive field center. Therefore, a pixel-type ganglion cell encoder represents a labeled line that signals a visual stimulus within its receptive field, and for downstream purposes the stimulus location with the highest probability is the cell’s receptive field center.

From here, first-order prediction is straightforward. When the image of a moving object of preferred contrast enters the receptive field periphery it will begin to depolarize the cell and potentially cause it to fire action potentials. While the object’s image moves from the receptive field perimeter toward the center, these stimulus-evoked action potentials may be transmitted down the optic nerve already before the object has reached the receptive field center. This is the essence of the predictive response and sets up the conditions for phase advanced motion encoding at the population level (Leonardo and Meister, 2013), and is what we observed for the majority of cells in our survey of mouse retinal ganglion cells.

Following early response onset, subsequent rapid shut-down of the initial action potential response through transient signaling enhances and solidifies the predictive signal, by shifting forward in space the center-of-mass of the ganglion cell population response (Leonardo and Meister, 2013). We believe that response transience through intrinsic spike adaptation is a key mechanism underlying phase advancement of response time to peak, as demonstrated by Leonardo and Meister (2013).

Cell-intrinsic spike adaptation has been demonstrated in transient-type ganglion cells. Most sustained response types, too, show some degree of spike adaptation where the initial response is greater than the sustained response phase (Zaghloul et al., 2005; Manookin and Demb, 2006). In both types (transient and sustained), spike adaptation can generate a meaningful phase-advanced response only if the cell’s response onset precedes the arrival of object’s image at the receptive field center. Here, we show that this is the case for the majority of the ganglion cells sampled in our dataset, and we find that this is true also for responses of the presynaptic bipolar cells. Our interpretation is that for biologically relevant speeds, spatial receptive field extent allows a cell to initiate depolarization before the image of a moving object reaches the receptive field center, thus effectively predicting the future position of the moving object. According to this working model, phase advancing should scale with receptive field size, and it does, as we show here: bipolar cells (small RFs) the least, α ganglion cells (large RFs) the most. This is consistent also with the study by Johnston and Lagnado (2015), where cells that failed to show PA had the smallest RF size.

### How reported mechanisms for phase advancing relate

The Leonardo/Meister model relies on an early response onset consistent with receptive field perimeter activation, followed by spike adaptation to maximize the forward shift of response time-to-peak. Our results show that many ganglion cell types as well as bipolar cell types in mouse show the early response onset that is required to shift forward the neuronal response to compensate for presynaptic signaling delay. While not all cells showed the transient response profile that would maximize the forward shift in position encoding at the cell population level, response transience at any subsequent signaling stage would generate a phase advanced population response, because the timing of response onset for visual object motion is already temporally advanced, as demonstrated here. While we did not test the spike or excitatory current adaptation model, our data show that response onset timing in most cells suffices for setting up a predictive, phase-advanced response.

Johnston and Lagnado (2015) showed that the phase-advanced response in goldfish retinal ganglion cells depends on feed-forward inhibition. The interpretation is that extra-classical receptive field interactions cause a moving object outside of the classical receptive field to release inhibition and so allow a cell to advance its response to a moving object as it approaches the receptive field center. Our pharmacological block of the major inhibitory neurotransmitter receptor types GABA and glycine did not cause a significant loss of phase advancing in the sampled ganglion cell types and bipolar cell types, indicating that the mechanism in mouse differs from that demonstrated in goldfish.

The Johnston and Lagnado (2015) study further showed that excitatory input to goldfish RGCs is delayed relative to the retinotopic position of a moving object, and that the transformation of this delayed signal to a phase advanced signal within each cell relies on nonlinear interaction of excitatory and inhibitory synaptic inputs. Our whole-cell voltage-clamp recordings from α-type ganglion cells do not agree with this, as we find that in these cells the excitatory signal is phase advanced (Fig. 3). Our finding appears consistent with the spatial “reach” of α-type ganglion cells, which collect excitatory synaptic input from bipolar cells ∼150 μm away, whose signals are already advanced by collecting synaptic input from cone photoreceptors up to 50 μm away from their receptive field centers. Whether these observed differences reflect species differences (goldfish vs mouse) or whether they may be specific to the particular ganglion cell types sampled in each study remains unclear.

### The role of gap junction coupling in phase advancing

An additional mechanism that could help generate a phase-advanced response are electrical gap junctions. Conceptually, electrical coupling between retinal cells of any type can spread activation laterally, priming neighboring cells along a moving stimulus trajectory to enhance their motion-evoked response. Gap junction coupling of one of four ON-OFF DSGC types was first demonstrated in rabbit retina (Vaney, 1994). The apparent homolog cell type in mouse retina is an upward-motion encoding DSGC labeled in the Hb9-eGFP transgenic line, and the response latency of Hb9 cells is, indeed, shorter than that of non-Hb9 DSGCs (Trenholm et al., 2013a). Electrical coupling of Hb9 cells creates an extensive subthreshold excitatory receptive field around each cell (Trenholm et al., 2013b) that enables electrically coupled cells to prime their downstream neighbors to fire earlier. This temporally advances the motion-evoked response and was also shown to correct for velocity-dependent spatial response lags (Trenholm et al., 2013a). Because our study did not identify upward-motion encoding DSGCs (see Results), it does not provide new information about possible differences in the phase advancing properties of this electrically-coupled DSGC type compared with apparent noncoupled DSGC types. Recent work in primate demonstrated that electrical coupling of retinal bipolar cells enabled predictive coding at the ganglion cell level by generating specific sensitivity to spatial correlations in incoming stimuli (Liu et al., 2021). Bipolar cells in mouse retina, too, have been shown to be electrically coupled, and therefore may contribute to the ganglion cells’ phase-advanced response through predictive coding.

### Phase advancing in bipolar cells

Glutamate imaging in the IPL (Fig. 6) showed that the onset of the bipolar cell synaptic response occurred before a moving spot reached the cell’s receptive field center. The motion-evoked response onset preceded the response evoked by a flashed spot, classifying the bipolar cell response as phase advanced. Our mechanistic explanation, like in the case of the ganglion cells, is that the bipolar cell spatial receptive field perimeter is located some 50 μm from its center. Depolarization from a moving spot crossing into the receptive field spreads across the entire axonal arbor and causes increased glutamate release throughout the axonal projective field, thus allowing synaptic transmission to start before the moving object’s image has reached the bipolar cell receptive field center (depending on object velocity and size). Our data show that for the velocities applied here, and a spot size that approximates the bipolar cell receptive field center, the magnitude of the advancement largely compensates for the signaling delay of phototransduction and signal transmission by the photoreceptors, i.e., it is large enough for the glutamate response to coincide with the spot crossing the bipolar cell receptive field center. In sum, we conclude that spatial integration in bipolar cells and ganglion cells aides visually-guided behavioral interactions with moving objects by countering neuronal signaling delays.

## Synthesis

Reviewing Editor: Susanne Wolf, Charite University Hospital Decisions are customarily a result of the Reviewing Editor and the peer reviewers coming together and discussing their recommendations until a consensus is reached. When revisions are invited, a fact-based synthesis statement explaining their decision and outlining what is needed to prepare a revision will be listed below. The following reviewer(s) agreed to reveal their identity: NONE. Note: If this manuscript was transferred from JNeurosci and a decision was made to accept the manuscript without peer review, a brief statement to this effect will instead be what is listed below.

Summary

The revised paper is a fair bit clearer and improved from the original submission. However, a few issues could still be clarified.

Major Comments

It remains unclear how informative the osPA measure is. Clearly RGCs and BCs have dendritic trees that extend laterally away from the soma (and RF center). Thus, it is rather trivial that a cell will initiate a response at quite a distance from the RF center during motion stimulus. I find it somewhat underwhelming that so much of the analytical focus is on this measurement, which more or less has to happen for any cell that can respond decently well to a moving stimulus. I would recommend de-emphasizing this measurement and maybe moving all these results to supplementary. Indeed, in the response to reviewers the authors acknowledge that the dendritic tree spread seems to account for the PA compensation for start time. If different RGC cell types could normalize the photoreceptor latency based on how their dendrites differentially integrate motion, then this point would be interesting, but the authors indicate that simply RGCs with larger dendritic trees have more osPA. I would therefore recommend instead focusing mostly on the phase advancement of time to peak, which seems to be the much more relevant feature.

Minor Comments

The focus of the paper, from its title and main discussion points, only focuses on phase advancing cells. However, the authors find that phase delay is also a common property (33% of OFF cells and 20% of ON cells). This seems worth emphasizing more openly in the discussion section. Why might the brain do this? Do the authors think these are specific types of cells that are not important for motion coding?

While I understand that the authors believe that their mechanisms arise from a combination of dendritic arbors reaching far away from the soma, combined with cell-intrinsic adaptation, it is a bit bizarre that they never so much as mention possible roles for gap junctions in the mechanism, or at least discuss gap junctions as another established method for lateral interactions (eg. Work from Awatramani lab in RGCs, and Rieke and Manookin labs in BCs).

## References

[B1] Baylor DA, Nunn BJ, Schnapf JL (1984) The photocurrent, noise and spectral sensitivity of rods of the monkey *Macaca fascicularis*. J Physiol 357:575–607. 10.1113/jphysiol.1984.sp015518 6512705 PMC1193276

[B2] Baylor DA, Nunn BJ, Schnapf JL (1987) Spectral sensitivity of cones of the monkey *Macaca fascicularis*. J Physiol 390:145–160. 3443931 10.1113/jphysiol.1987.sp016691 PMC1192171

[B3] Berry MJ, Brivanlou IH, Jordan TA, Meister M (1999) Anticipation of moving stimuli by the retina. Nature 398:334–338. 10.1038/18678 10192333

[B4] Borghuis BG, Leonardo A (2015) The role of motion extrapolation in amphibian prey capture. J Neurosci 35:15430–15441. 10.1523/JNEUROSCI.3189-15.2015 26586829 PMC4649011

[B5] Borghuis BG, Tian L, Xu Y, Nikonov SS, Vardi N, Zemelman BV, Looger LL (2011) Imaging light responses of targeted neuron populations in the rodent retina. J Neurosci 31:2855–2867. 10.1523/JNEUROSCI.6064-10.2011 21414907 PMC3521507

[B6] Borghuis BG, Marvin JS, Looger LL, Demb JB (2013) Two-photon imaging of nonlinear glutamate release dynamics at bipolar cell synapses in the mouse retina. J Neurosci 33:10972–10985. 10.1523/JNEUROSCI.1241-13.2013 23825403 PMC3718381

[B7] Brainard DH (1997) The psychophysics toolbox. Spat Vis 10:433–436. 10.1163/156856897X00357 9176952

[B8] Cafaro J, Zylberberg J, Field GD (2020) Global motion processing by populations of direction-selective retinal ganglion cells. J Neurosci 40:5807–5819. 10.1523/JNEUROSCI.0564-20.2020 32561674 PMC7380974

[B9] Chen TW, Wardill TJ, Sun Y, Pulver SR, Renninger SL, Baohan A, Schreiter ER, Kerr RA, Orger MB, Jayaraman V, Looger LL, Svoboda K, Kim DS (2013) Ultrasensitive fluorescent proteins for imaging neuronal activity. Nature 499:295–300. 10.1038/nature12354 23868258 PMC3777791

[B10] Chichilnisky EJ (2001) A simple white noise analysis of neuronal light responses. Network 12:199–213. 10.1080/713663221 11405422

[B11] Dana H, Chen T-W, Hu A, Shields BC, Guo C, Looger LL, Kim DS, Svoboda K (2014) Thy1-GCaMP6 transgenic mice for neuronal population imaging in vivo. PLoS One 9:e108697. 10.1371/journal.pone.0108697 25250714 PMC4177405

[B12] Demb JB, Haarsma L, Freed MA, Sterling P (1999) Functional circuitry of the retinal ganglion cell’s nonlinear receptive field. J Neurosci 19:9756–9767. 10.1523/JNEUROSCI.19-22-09756.1999 10559385 PMC6782950

[B14] Fransen JW, Borghuis BG (2017) Temporally diverse excitation generates direction-selective responses in ON- and OFF-type retinal starburst amacrine cells. Cell Rep 18:1356–1365. 10.1016/j.celrep.2017.01.026 28178515

[B15] Gollisch T, Meister M (2008) Rapid neural coding in the retina with relative spike latencies. Science 319:1108–1111. 10.1126/science.1149639 18292344

[B18] Jacoby J, Schwartz GW (2017) Three small-receptive-field ganglion cells in the mouse retina are distinctly tuned to size, speed, and object motion. J Neurosci 37:610–625. 10.1523/JNEUROSCI.2804-16.2016 28100743 PMC5242408

[B19] Jancke D, Erlhagen W, Schöner G, Dinse HR (2004) Shorter latencies for motion trajectories than for flashes in population responses of cat primary visual cortex. J Physiol 556:971–982. 10.1113/jphysiol.2003.058941 14978201 PMC1665003

[B20] Johnson KP, Fitzpatrick MJ, Zhao L, Wang B, McCracken S, Williams PR, Kerschensteiner D (2021) Cell-type-specific binocular vision guides predation in mice. Neuron 109:1527–1539.e4. 10.1016/j.neuron.2021.03.010 33784498 PMC8112612

[B21] Johnston J, Lagnado L (2015) General features of the retinal connectome determine the computation of motion anticipation. Elife 4:e06250. 10.7554/eLife.06250 PMC4391023 25786068

[B22] Krieger B, Qiao M, Rousso DL, Sanes JR, Meister M (2017) Four alpha ganglion cell types in mouse retina: function, structure, and molecular signatures. PLoS One 12:e0180091. 10.1371/journal.pone.0180091 28753612 PMC5533432

[B23] Leonardo A, Meister M (2013) Nonlinear dynamics support a linear population code in a retinal target-tracking circuit. J Neurosci 33:16971–16982. 10.1523/JNEUROSCI.2257-13.2013 24155302 PMC3807026

[B24] Liu B, Hong A, Rieke F, Manookin MB (2021) Predictive encoding of motion begins in the primate retina. Nat Neurosci 24:1280–1291. 10.1038/s41593-021-00899-1 34341586 PMC8728393

[B25] Manookin MB, Demb JB (2006) Presynaptic mechanism for slow contrast adaptation in mammalian retinal ganglion cells. Neuron 50:453–464. 10.1016/j.neuron.2006.03.039 16675399

[B26] Manookin MB, Beaudoin DL, Ernst ZR, Flagel LJ, Demb JB (2008) Disinhibition combines with excitation to extend the operating range of the OFF visual pathway in daylight. J Neurosci 28:4136–4150. 10.1523/JNEUROSCI.4274-07.2008 18417693 PMC2557439

[B27] Marvin JS, Borghuis BG, Tian L, Cichon J, Harnett MT, Akerboom J, Gordus A, Renninger SL, Chen TW, Bargmann CI, Orger MB, Schreiter ER, Demb JB, Gan WB, Hires SA, Looger LL (2013) An optimized fluorescent probe for visualizing glutamate neurotransmission. Nat Methods 10:162–170. 10.1038/nmeth.2333 23314171 PMC4469972

[B28] Mearns DS, Donovan JC, Fernandes AM, Semmelhack JL, Baier H (2020) Deconstructing hunting behavior reveals a tightly coupled stimulus-response loop. Curr Biol 30:54–69.e9. 10.1016/j.cub.2019.11.022 31866365

[B29] Mischiati M, Lin HT, Herold P, Imler E, Olberg R, Leonardo A (2015) Internal models direct dragonfly interception steering. Nature 517:333–338. 10.1038/nature14045 25487153

[B30] Mysore SP, Asadollahi A, Knudsen EI (2010) Global inhibition and stimulus competition in the owl optic tectum. J Neurosci 30:1727–1738. 10.1523/JNEUROSCI.3740-09.2010 20130182 PMC2828882

[B31] Nobles RD, Zhang C, Müller U, Betz H, McCall MA (2012) Selective glycine receptor α2 subunit control of crossover inhibition between the ON and OFF retinal pathways. J Neurosci 32:3321–3332. 10.1523/JNEUROSCI.5341-11.2012 22399754 PMC3438913

[B32] Pang JJ, Gao F, Wu SM (2003) Light-evoked excitatory and inhibitory synaptic inputs to ON and OFF alpha ganglion cells in the mouse retina. J Neurosci 23:6063–6073. 10.1523/JNEUROSCI.23-14-06063.2003 12853425 PMC6740343

[B33] Pologruto TA, Sabatini BL, Svoboda K (2003) ScanImage: flexible software for operating laser scanning microscopes. Biomed Eng Online 2:13. 10.1186/1475-925X-2-13 12801419 PMC161784

[B34] Sakai HM (1992) White-noise analysis in neurophysiology. Physiol Rev 72:491–505. 10.1152/physrev.1992.72.2.491 1557430

[B35] Schnapf JL, Kraft TW, Baylor DA (1987) Spectral sensitivity of human cone photoreceptors. Nature 325:439–441. 10.1038/325439a0 3808045

[B36] Schwartz G, Taylor S, Fisher C, Harris R, Berry MJ 2nd (2007) Synchronized firing among retinal ganglion cells signals motion reversal. Neuron 55:958–969. 10.1016/j.neuron.2007.07.042 17880898 PMC3163230

[B37] Schwartz GW, Okawa H, Dunn FA, Morgan JL, Kerschensteiner D, Wong RO, Rieke F (2012) The spatial structure of a nonlinear receptive field. Nat Neurosci 15:1572–1580. 10.1038/nn.3225 23001060 PMC3517818

[B39] Trenholm S, Schwab DJ, Balasubramanian V, Awatramani GB (2013a) Lag normalization in an electrically coupled neural network. Nat Neurosci 16:154–156. 10.1038/nn.3308 23313908

[B40] Trenholm S, McLaughlin AJ, Schwab DJ, Awatramani GB (2013b) Dynamic tuning of electrical and chemical synaptic transmission in a network of motion coding retinal neurons. J Neurosci 33:14927–14938. 10.1523/JNEUROSCI.0808-13.2013 24027292 PMC6705162

[B41] Tsukamoto Y, Omi N (2017) Classification of mouse retinal bipolar cells: type-specific connectivity with special reference to rod-driven AII amacrine pathways. Front Neuroanat 11:92–92. 10.3389/fnana.2017.00092 29114208 PMC5660706

[B42] Turner MH, Schwartz GW, Rieke F (2018) Receptive field center-surround interactions mediate context-dependent spatial contrast encoding in the retina. Elife 7:e38841. 10.7554/eLife.38841 30188320 PMC6185113

[B43] Vaney DI (1994) Territorial organization of direction-selective ganglion cells in rabbit retina. J Neurosci 14:6301–6316. 10.1523/JNEUROSCI.14-11-06301.1994 7965037 PMC6577224

[B44] Van Wyk M, Wässle H, Taylor WR (2009) Receptive field properties of ON- and OFF-ganglion cells in the mouse retina. Vis Neurosci 26:297–308. 10.1017/S0952523809990137 19602302 PMC2874828

[B45] Wardill TJ, Fabian ST, Pettigrew AC, Stavenga DG, Nordström K, Gonzalez-Bellido PT (2017) A novel interception strategy in a miniature robber fly with extreme visual acuity. Curr Biol 27:854–859. 10.1016/j.cub.2017.01.050 28286000 PMC5364399

[B46] Zaghloul KA, Boahen K, Demb JB (2005) Contrast adaptation in subthreshold and spiking responses of mammalian Y-type retinal ganglion cells. J Neurosci 25:860–868. 15673666 10.1523/JNEUROSCI.2782-04.2005 PMC6725633

[B47] Zhang Y, Kim I-J, Sanes JR, Meister M (2012) The most numerous ganglion cell type of the mouse retina is a selective feature detector. Proc Natl Acad Sci U S A 109:E2391–E2398. 10.1073/pnas.1211547109 22891316 PMC3437843

