# Supplementary figures and images for: Phase Advancing Is a Common Property of Multiple Neuron Classes in the Mouse Retina
Source: eNeuro. 2022 Sep 1;9(5):ENEURO.0270-22.2022. doi: 10.1523/ENEURO.0270-22.2022 (PMC9450563; doi:10.1523/ENEURO.0270-22.2022)

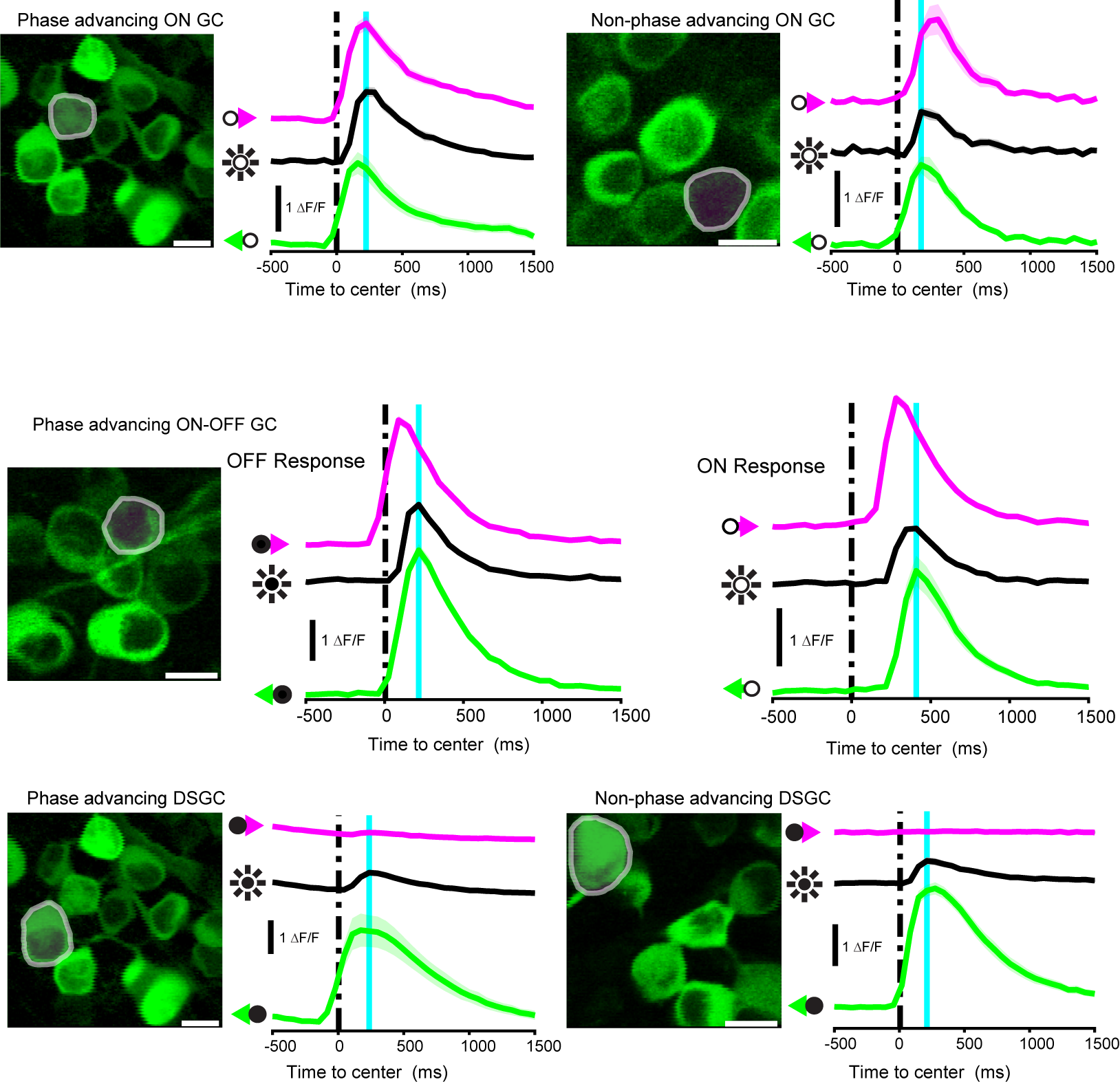

Supplement: Extended Data Figure 2-1 — Optical recordings show phase-advanced responses in diverse ganglion cell populations. Two-photon fluorescence images of GCaMP6f-expressing cells in the ganglion cell layer (left) and accompanying GCaMP6f fluorescence responses for the indicated cell (gray circle). Scale bar = 10 μm. The vertical dashed black line indicates t0, i.e., when the spot crossed the center of the imaging window; vertical cyan line shows the peak response time for the flashed spot. We measured phase-advanced responses in all functionally defined ganglion cell populations shown here (ON, ON-OFF non-DS, and DS). Download Figure 2-1, TIF file. [file enu-eN-NWR-0270-22-s01.tif]

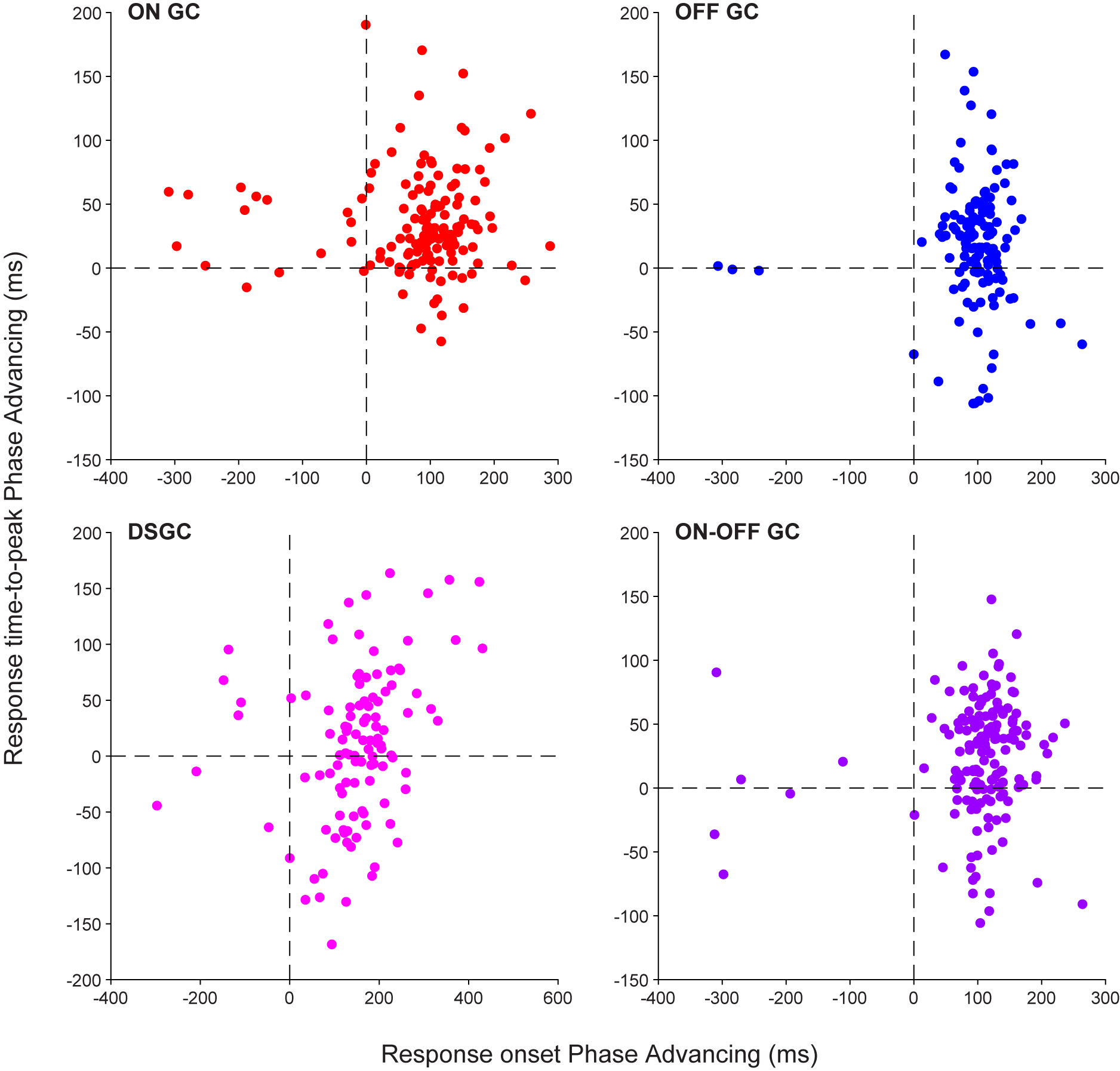

Supplement: Extended Data Figure 2-2 — Scatter plots of response onset versus response time-to-peak phase advancing for all individual cells identified in the GCaMP6f population imaging experiments (ON GCs: n = 171; OFF GCs: n = 180; DSGCs: n = 157; ON-OFF GCs: n = 202). Download Figure 2-2, TIF file. [file enu-eN-NWR-0270-22-s02.tif]

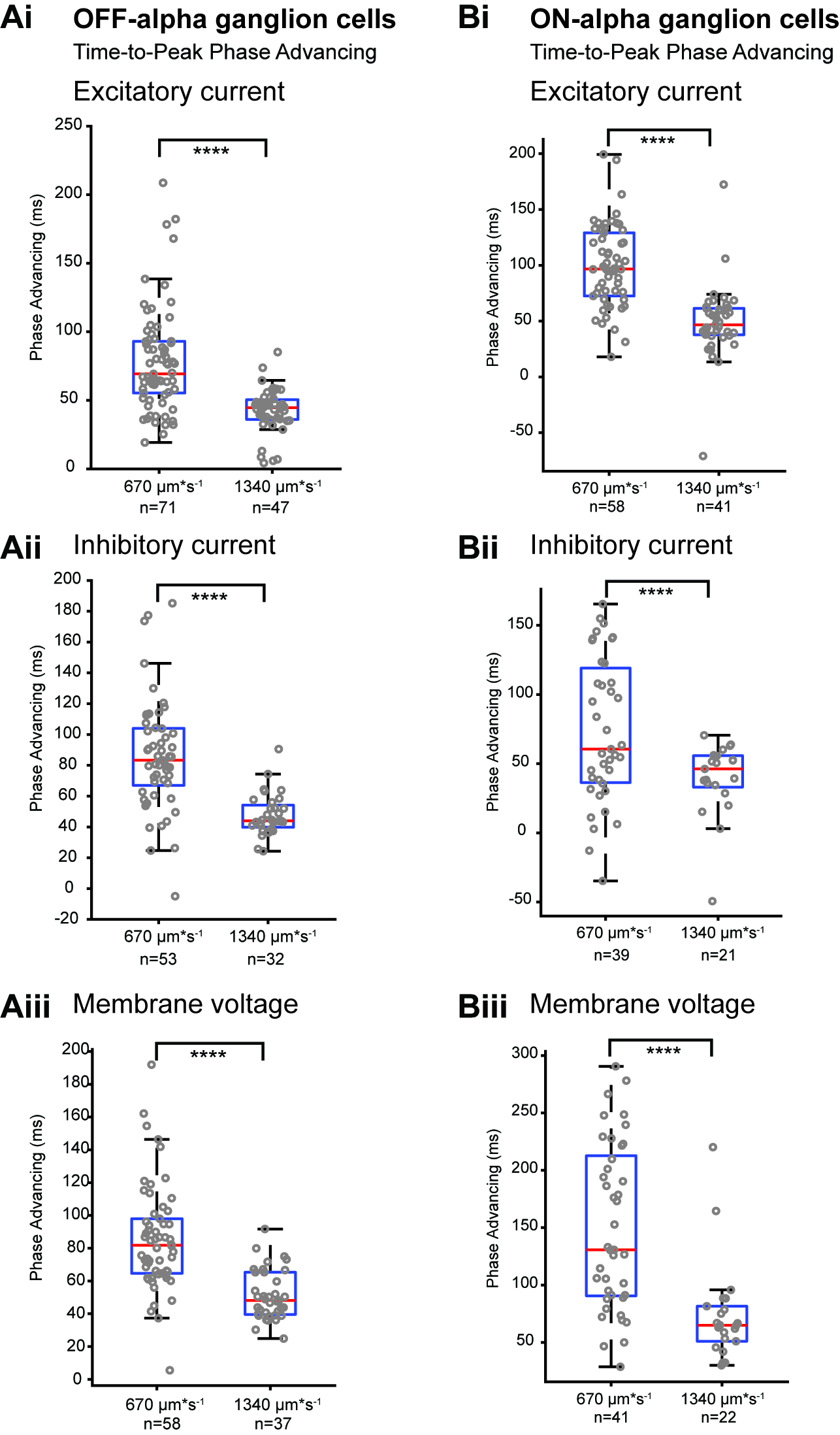

Supplement: Extended Data Figure 3-1 — A, Response time-to-peak phase advance values of OFF-α ganglion cells measured using electrophysiological whole-cell recordings at the level of the excitatory current (i), inhibitory current (ii), and membrane voltage (iii). The stimulus set comprised spots moving at two velocities. Faster moving spots gave smaller time-to-peak phase advance values. B, As A, for ON-α ganglion cells. Download Figure 3-1, TIF file. [file enu-eN-NWR-0270-22-s04.tif]

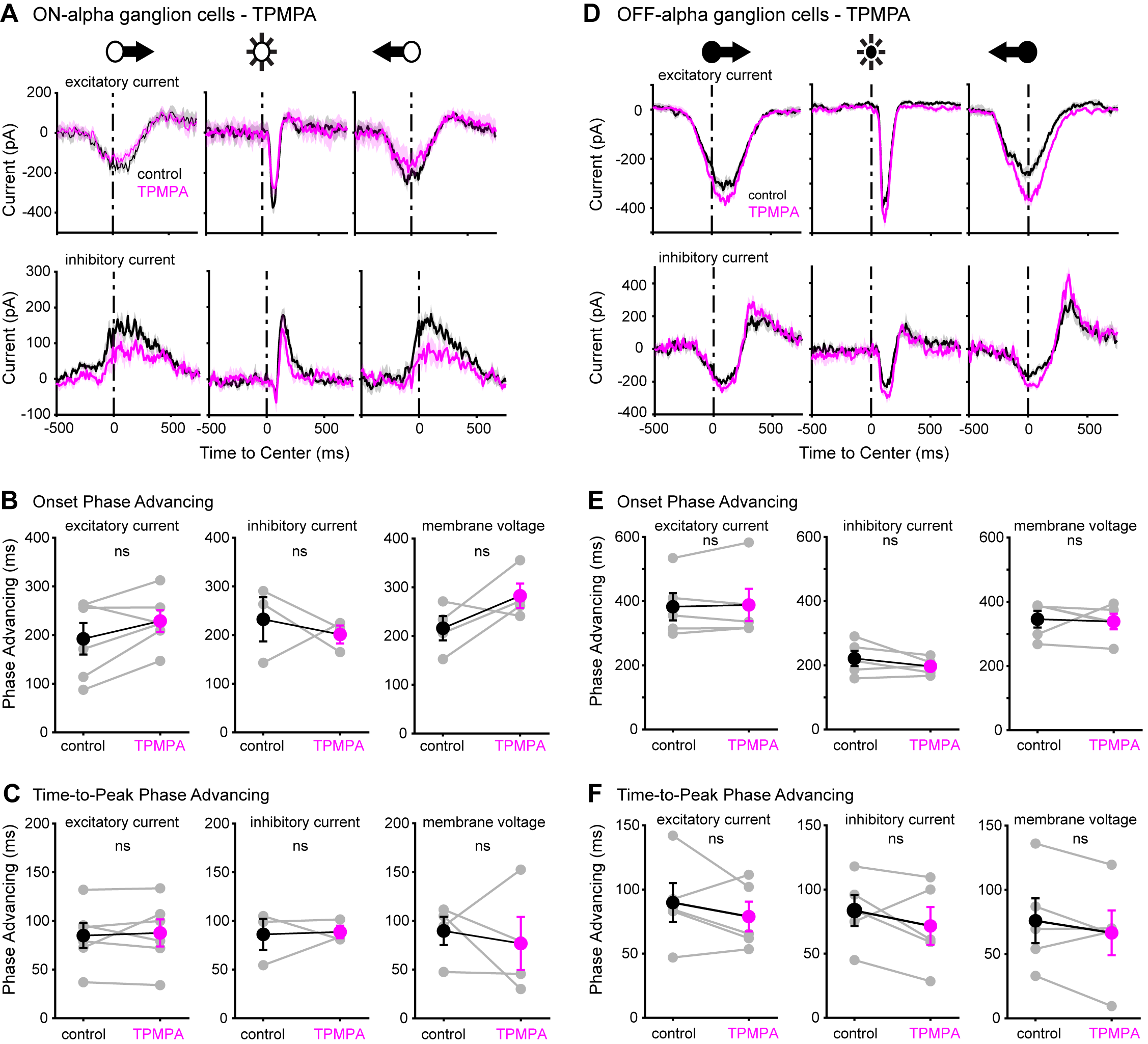

Supplement: Extended Data Figure 5-1 — Pharmacological block of GABAa-rho receptors does not alter response timing in α-type ganglion cells. A, Excitatory (top) and inhibitory (bottom) currents obtained using electrophysiological whole-cell recording from an ON-α ganglion cell under control conditions (black) and in the presence of selective GABAa-rho (former GABAc) receptor blocker TPMPA (50 μm; magenta). B, Response onset phase advancing for the recorded ON-α ganglion cell population (n = 6). C, Response time-to-peak phase advancing for the recorded ON-α ganglion cell population. D–F, as A–C, for OFF-α ganglion cells (n = 5). Download Figure 5-1, TIF file. [file enu-eN-NWR-0270-22-s05.tif]

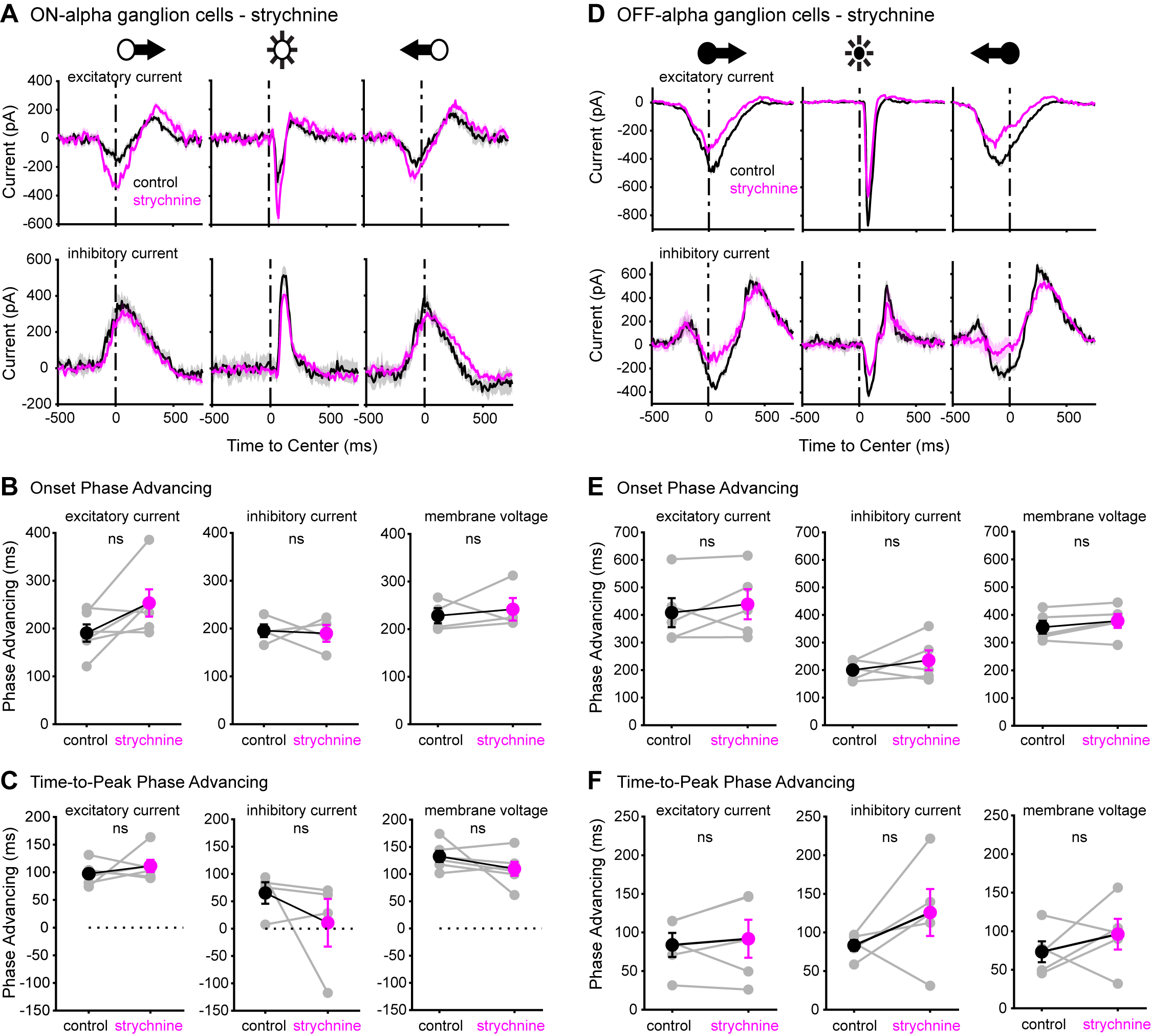

Supplement: Extended Data Figure 5-2 — Pharmacological block of glycine receptors does not alter response timing in α-type ganglion cells. A, Excitatory (top) and inhibitory (bottom) currents obtained using electrophysiological whole-cell recording from an ON-α ganglion cell under control conditions (black) and in the presence of glycine receptor blocker strychnine (1 μm; magenta). B, Response onset phase advancing for the recorded ON-α ganglion cell population (n = 6). C, Response time-to-peak phase advancing for the recorded ON-α ganglion cell population. D–F, As A–C, for OFF-α ganglion cells (n = 5). Download Figure 5-2, TIF file. [file enu-eN-NWR-0270-22-s06.tif]
